# DnaB helicase dynamics in bacterial DNA replication resolved by single-molecule studies

**DOI:** 10.1093/nar/gkab493

**Published:** 2021-06-17

**Authors:** Richard R Spinks, Lisanne M Spenkelink, Sarah A Stratmann, Zhi-Qiang Xu, N Patrick J Stamford, Susan E Brown, Nicholas E Dixon, Slobodan Jergic, Antoine M van Oijen

**Affiliations:** Molecular Horizons and School of Chemistry and Molecular Bioscience, University of Wollongong, Wollongong, New South Wales 2522, Australia; Illawarra Health & Medical Research Institute, Wollongong, New South Wales 2522, Australia; Molecular Horizons and School of Chemistry and Molecular Bioscience, University of Wollongong, Wollongong, New South Wales 2522, Australia; Illawarra Health & Medical Research Institute, Wollongong, New South Wales 2522, Australia; Zernike Institute for Advanced Materials, University of Groningen, Groningen 9747 AG, The Netherlands; Molecular Horizons and School of Chemistry and Molecular Bioscience, University of Wollongong, Wollongong, New South Wales 2522, Australia; Illawarra Health & Medical Research Institute, Wollongong, New South Wales 2522, Australia; Research School of Chemistry, Australian National University, Canberra, Australian Capital Territory 2601, Australia; Research School of Chemistry, Australian National University, Canberra, Australian Capital Territory 2601, Australia; Molecular Horizons and School of Chemistry and Molecular Bioscience, University of Wollongong, Wollongong, New South Wales 2522, Australia; Illawarra Health & Medical Research Institute, Wollongong, New South Wales 2522, Australia; Research School of Chemistry, Australian National University, Canberra, Australian Capital Territory 2601, Australia; Molecular Horizons and School of Chemistry and Molecular Bioscience, University of Wollongong, Wollongong, New South Wales 2522, Australia; Illawarra Health & Medical Research Institute, Wollongong, New South Wales 2522, Australia; Molecular Horizons and School of Chemistry and Molecular Bioscience, University of Wollongong, Wollongong, New South Wales 2522, Australia; Illawarra Health & Medical Research Institute, Wollongong, New South Wales 2522, Australia

## Abstract

In *Escherichia coli*, the DnaB helicase forms the basis for the assembly of the DNA replication complex. The stability of DnaB at the replication fork is likely important for successful replication initiation and progression. Single-molecule experiments have significantly changed the classical model of highly stable replication machines by showing that components exchange with free molecules from the environment. However, due to technical limitations, accurate assessments of DnaB stability in the context of replication are lacking. Using *in vitro* fluorescence single-molecule imaging, we visualise DnaB loaded on forked DNA templates. That these helicases are highly stable at replication forks, indicated by their observed dwell time of ∼30 min. Addition of the remaining replication factors results in a single DnaB helicase integrated as part of an active replisome. In contrast to the dynamic behaviour of other replisome components, DnaB is maintained within the replisome for the entirety of the replication process. Interestingly, we observe a transient interaction of additional helicases with the replication fork. This interaction is dependent on the τ subunit of the clamp-loader complex. Collectively, our single-molecule observations solidify the role of the DnaB helicase as the stable anchor of the replisome, but also reveal its capacity for dynamic interactions.

## Graphical Abstract

**Graphical Abstract:**
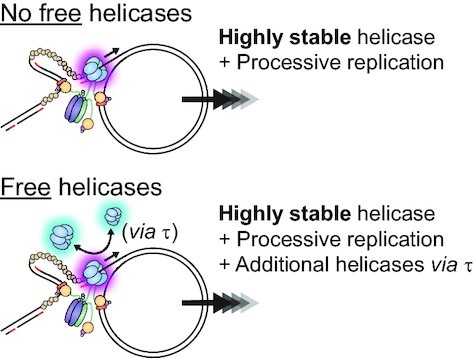
Single-molecule observation of the DnaB helicase in *E. coli* DNA replication shows a single, stably-integrated helicase supports replication, with additional helicases able to interact via available τ subunits.

## INTRODUCTION

**Figure 1.**
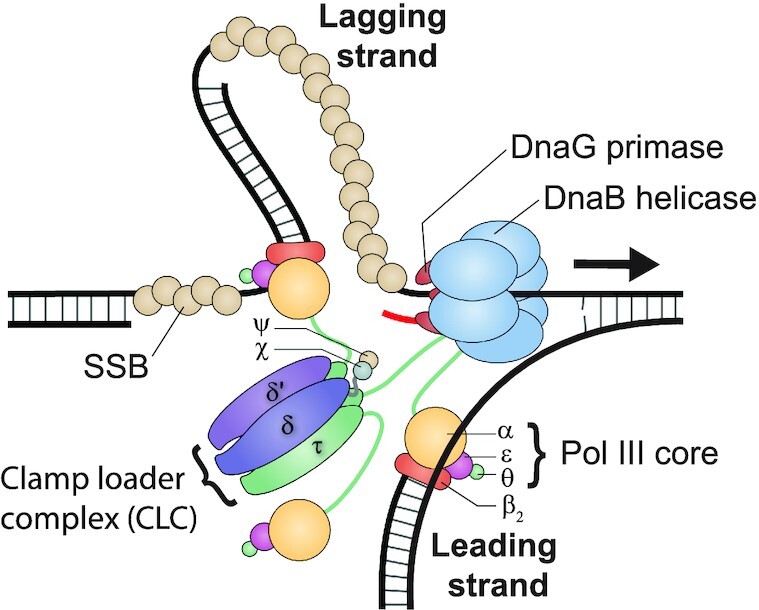
Schematic representation of the architecture of the *E. coli* replisome. The DnaB helicase enables the progression of the replisome as it unwinds double-stranded DNA. DnaG primase synthesises short RNA primers (shown in red) on the single-stranded DNA template. The clamp-loader complex (CLC; consisting of δ, δ', ψ, χ and three τ) loads the β_2_ sliding clamp and the αϵθ polymerase III core onto newly primed sites. The core then synthesises new DNA on both template strands. DNA synthesis occurs continuously on the leading strand and discontinuously on the lagging strand. The CLC tethers the polymerase to the helicase via one τ subunit. Single-stranded DNA-binding protein (SSB) coats and protects the transiently exposed DNA on the lagging strand.

All living organisms depend on the accurate duplication of their genomic DNA to ensure their survival. In every domain of life, this intricate process is fulfilled by a set of co-evolved enzymes collectively known as the replisome. In the model bacterium *Escherichia coli*, the replisome (Figure 1) consists of twelve separate proteins that assemble on DNA and combine their actions to faithfully copy the circular chromosome (reviewed in (1)). The replicative helicase DnaB plays a key role within the replisome. It is a homo-hexameric, RecA-like ATPase (2) that encircles single-stranded (ss) DNA, on which it hydrolyses ATP or other rNTPs to drive its translocation in the 5′–3′ direction. DnaB unwinds the parental double-stranded (ds) DNA and thereby provides the ssDNA templates for DNA polymerase and primase activity. During replication initiation, the loading of DnaB at the replication origin (*oriC*) is observed as the key determinant in replisome assembly (reviewed in (3)). In order to be loaded, DnaB must form a tight complex with its helicase-loader protein DnaC, and then be recruited to *oriC* by the DnaA initiator protein. The DnaC helicase loader destabilises the DnaB hexamer and forms with it a three-tiered, right-handed cracked-ring structure that enables deposition of the helicase onto ssDNA (4–6). Once loaded, the hexameric helicase presents sites for up to two DnaG primase molecules to interact, leading to subsequent ejection of DnaC (7). During replication, two or three helicase-bound primase molecules cooperate to produce short RNA primers required for initiation of DNA polymerase activity (8,9). The DNA polymerase III holoenzyme (Pol III HE) is the replicase responsible for engaging the primers on both the leading and lagging strands and extending them into new DNA. Pol III HE consists of three sub-assemblies: the αϵθ polymerase III core, the clamp loader complex (CLC) and the β_2_ processivity clamp (10). The CLC hydrolyses ATP to load the β_2_ clamp onto DNA, which in turn stabilises the Pol III core while it synthesises DNA (11–13). The CLC also acts as the physical bridge between the Pol III cores and DnaB helicase through its multiple τ subunits (14–16).

The *E. coli* replisome is able to duplicate the entire 4.6 Mb chromosome with high speed and accuracy (17,18). Due to the complex network of strong and weak interactions within the replisome, the nature of the interactions and how they permit the replisome to coordinate its various enzymatic activities are still not fully understood. The DnaB helicase, however, is generally acknowledged as the key for orchestrating the formation of the multi-enzyme replisomal complex at the replication fork. Consequently, the stability of the replicative helicase at the fork is likely an important parameter underlying successful replication initiation and processive fork progression. To date, DnaB stability during replication has only been substantiated to a limited extent through studies into DnaB binding kinetics, unwinding analysis and helicase structures in various states (reviewed in (19)).

*In vivo* single-molecule studies have characterised the average stoichiometry of DnaB at the fork (20–22). One of these studies estimates the lifetime of DnaB at the fork to be on the order of 15 min (22). In a similar scenario, several ensemble *in vivo* studies of replication fork stalling infer the stability of DnaB after stalling to be between 5 and 30 min (23–26). Due to the nature of these *in vivo* methods, technical limitations prevent accurate detection of DnaB stability. It is clear that DnaB remains at the fork longer than other replisome components, but the exact stability of the helicase during loading and replication is still uncertain.

Recently, single-molecule studies have revealed the ability of many replisome components to exchange with equivalent molecules in the surrounding environment (22,27–32). Specifically, the *E. coli* replicative polymerases were shown to rapidly exchange in a concentration-dependent manner (31). Similar behaviour was observed for the single-stranded DNA-binding protein (SSB) that is carried with the replisome (29). This emerging picture of plasticity in DNA replication portrays the replisome as a multi-faceted machine capable of sampling parallel reaction pathways to fulfil its goal (33,34). In light of these observations of the rapid dynamics of many replisome components, it could be inferred that the integrity of the replisome is linked to the stability of the DnaB helicase. It is tempting to speculate about an alternative scenario—that DnaB might also have the capability for dynamic exchange, and thus the entire replisome would be in a constant state of flux.

To assess the stability of the DnaB helicase in the context of replication, we use single-molecule assays to directly visualise individual helicases during DNA replication. In an *in vitro* reconstituted system, we use fluorescently labelled DnaB to allow us to monitor helicase behaviour in real time. We find that the active DnaB helicase remains stably associated even in the presence of a large excess of free DnaB molecules. Interestingly, additional helicases do transiently interact with the replisome. This transient interaction is enabled through the τ subunit of Pol III HE. In contrast to the dynamic behaviour of other replisome components, our results explicitly demonstrate that DnaB acts as a stable anchor within the replisome, thereby providing the interaction platform necessary to maintain replisome integrity throughout the processive replication of the bacterial chromosome.

## MATERIALS AND METHODS

### Replication proteins

*Escherichia coli* DNA replication proteins were produced from *E. coli* expression strains using genes from *E. coli* K12 strains as described previously: the β_2_ sliding clamp (35); SSB (36); DnaG primase (37); Pol III αϵθ core (31); and the Pol III clamp loader complexes τ_3_δδ’ψχ, τ_2_γ_1_δδ’ψχ, τ_1_γ_2_δδ’ψχ (38), and γ_3_δδ’ψχ (12). DnaB_6_, DnaC and the DnaB_6_(DnaC)_6_ helicase–loader complex were produced through a new and more effective method. The overexpression and purification steps of this method are detailed in Supplementary Methods and Supplementary Figure S1. Concentrations of DnaB_6_, DnaC and the DnaB_6_(DnaC)_6_ complex were determined using the Quick Start Bradford Dye Reagent (Bio-Rad).

### Production of DnaB_6_-H201C

The *dnaB* H201C mutation (Supplementary Figure S2) was made by a two-step overlap extension PCR, with internal mutagenic primers 578/H201C/F (5′-GCAGCCATGCGATGGCGTTACCGGGG) and 579/H201C/R (5′-GCCATCGCATGGCTGCTGAAACAACTG) and external primers 299 (5′-TGGGTGATCTTCAACTGG) and 300 (5′-TGTTCACGGGCAATACG). Plasmid DNA of pSB958 encoding wild-type DnaB (Supplementary Figure S1) was used as template; this plasmid contains a synthetic *E. coli dnaCB* operon in a pCE30 derivative (39), with a unique NdeI site at the start codon of *dnaB*. Primer 299 is located upstream of the *dnaB* gene and 300 is inside *dnaB*, beyond a unique NcoI site. The final PCR product was cleaned up using a QIAquick PCR Purification Kit (Qiagen), digested with NdeI and NcoI, and the digested product used to replace the corresponding NdeI–NcoI fragment of pSB958. The nucleotide sequence of the inserted fragment in the resulting plasmid pZX1548 confirmed the presence of the H201C mutation and the absence of other mutations. Expression and purification of DnaB_6_-H201C was carried out in the same manner as the wild-type DnaB_6_ helicase (Supplementary Methods).

### Labelling of DnaB_6_-H201C

The labelling methods described are based on a published procedure (40). DnaB_6_-H201C was labelled with Alexa Fluor 647 (Invitrogen) by solid-state labelling followed by multiple rounds of ammonium sulphate washing. First, 2 mg of DnaB_6_-H201C was reduced by adding 15 mM additional fresh dithiothreitol (DTT) to the existing buffer. The protein was then precipitated by gradual addition of solid ammonium sulphate (0.45 g ml^–1^), centrifuged (21 000 × *g*; 20 min) and the supernatant carefully removed. The protein was washed with 1 ml ice-cold labelling buffer (30 mM Tris–HCl pH 6.8, 100 mM NaCl, 0.5 mM EDTA, 10 mM MgCl_2_, 100 mM ADP) + 70% (w/v) ammonium sulphate that is now devoid of reducing agent and has been extensively degassed by sonication and deoxygenated with N_2_ gas. The protein was centrifuged again (21 000 × *g*; 20 min) and the pellet resuspended in 1 ml labelling buffer + 70% (w/v) ammonium sulphate + 0.95 mg AF647-maleimide dye (dissolved in 30 μl DMSO immediately before use; 20:1 molar ratio of dye to cysteine). The reaction was allowed to proceed by rotating gently overnight at 6°C in the dark. DnaB_6_-H201C–AF647 was separated from excess dye by centrifugation of the solution (21 000 × *g*; 20 min) and then washing the protein with 1 ml labelling buffer + 70% (w/v) ammonium sulphate + 4 mM DTT. The wash steps were repeated 15 times until no more dye remained in the supernatant. DnaB_6_–H201C–AF647 was dialysed into storage buffer (labelling buffer + 4 mM DTT, 20% v/v glycerol). The purity was confirmed by SDS-PAGE and the degree of labelling was determined by to be 3–4 dyes per DnaB hexamer by detection of single-molecule photobleaching steps (see Data analysis).

DnaB_6_–H201C was also labelled with Alexa Fluor 488 (Invitrogen) by solid-state labelling followed by column chromatography and ammonium sulphate washing (procedure modified to improve protein yield). The labelling procedure was the same as above until the purification steps. After the reaction was complete, DnaB_6_–H201C–AF488 was separated from excess dye by centrifugation (21 000 × *g*; 20 min) and resuspended in 1 ml storage buffer. To remove the remaining dye, the labelled protein was applied at 1 ml min^–1^ to a column (1.5 × 10 cm) of Sephadex G-25 (GE Healthcare) equilibrated in storage buffer. Fractions containing DnaB_6_–H201C–AF488 were pooled, purity confirmed by SDS-PAGE (Supplementary Figure S3) and the degree of labelling determined to be 2–3 dyes per DnaB hexamer as above.

### Ensemble replication assay of DnaB activity

Both Alexa Fluor 647- and Alexa Fluor 488-labelled DnaB_6_, now referred to as DnaB_6_(red) and DnaB_6_(blue) respectively, were tested for their activity in a bulk solution-phase leading-strand replication assay measuring the duplication of a flap-primed single-stranded M13 DNA template (31). First, the primed ssDNA template was made by annealing M13mp18 ssDNA (Guild BioSciences) with a 66-mer (IDT) oligonucleotide consisting of a 30-nt complementary segment. Each replication reaction contained 2.5 nM primed DNA template, 90 nM αϵθ core, 30 nM τ_3_δδ’ψχ clamp loader, 200 nM β_2_ clamp, 60 nM DnaB_6_(red) or DnaB_6_(blue) and 315 nM DnaC in bulk replication buffer (30 mM Tris–HCl pH 7.6, 12 mM magnesium acetate, 50 mM potassium glutamate, 0.5 mM EDTA, 0.0025% v/v Tween20, 1 mM ATP, 10 mM DTT, 100 μM of each dNTP) in a final volume of 10 μl. All the reaction components were mixed (except for DNA) and cooled on ice before the reaction was initiated by adding DNA and moving to 30°C. After 10 min the reaction was stopped by addition of 10 μl heated quench buffer (200 mM EDTA, 2% w/v SDS, 1× DNA loading dye mix). The quenched mixtures were loaded into a 0.7% (w/v) agarose gel in TAE running buffer (160 mM Tris base pH 8.2, 80 mM acetic acid, 2 mM EDTA). DNA products were separated by electrophoresis for 100 min at 75 V, then stained with SYBR Gold and visualised by UV light on a Gel Doc XR (Bio-Rad).

Using this ensemble replication assay, DnaB_6_(red) and DnaB_6_(blue) were shown to support DNA replication as compared to WT DnaB_6_ and inactive DnaB_6_ (inactive DnaB_6_ comes from a bad preparation of this protein) (Supplementary Figure S3). Each labelled DnaB helicase proved capable of promoting DNA replication.

### Single-molecule DnaB-loading assay

Microfluidic flow-cell devices were prepared as previously described (41,42). Briefly, a flow chamber was created by adhering a PDMS block with an indented channel to a functionalised biotin (Laysan Bio) coverslip. To reduce non-specific interactions with the surface during the experiment, the flow chamber was injected with degassed blocking buffer (50 mM Tris–HCl pH 7.6, 100 mM NaCl, 2% w/v Tween20) and incubated for 30–60 min. The flow-cell device was mounted on an inverted TIRF microscope (Nikon Eclipse Ti-E), with an electrically heated stage (31°C; Okolab), a 100x TIRF objective (NA = 1.49, oil, Nikon), and connected syringe pump (Adelab Scientific).

The conditions used to measure DnaB loading were modified from previously described single-molecule replication experiments (29,31,38,42). Construction of a 2030-bp rolling-circle DNA template with controlled fork topology has been described previously (43). The loading solution contained 4 nM DnaB_6_(red), 21 nM DnaC and 20 pM rolling-circle DNA template (components preloaded *in situ* by incubating in 20 μl for 5 min at 37°C) in 150 μl degassed single-molecule loading buffer (30 mM Tris–HCl pH 7.6, 12 mM magnesium acetate, 50 mM potassium glutamate, 0.5 mM EDTA, 0.0025% w/v Tween20, 0.5 mg ml^–1^ BSA, 1 mM ATP, 10 mM DTT, 150 nM SYTOX Orange (Invitrogen)). The loading solution was injected into the blocked flow chamber at 100 μl min^−1^ for 1 min and then at 5 μl min^−1^ for 10 min.

To detect DnaB loaded onto DNA, the SYTOX Orange-stained rolling-circle DNA template was visualised in real time with either a 514-nm (Coherent, Sapphire 514-150 CW) or 568-nm laser (Coherent, Sapphire 568-200 CW) at 400 mW cm^−2^ for 1 s once every 30 s. The DnaB_6_(red) protein was visualised by constant excitation with a 647-nm laser (Coherent, Obis 647-100 CW) at 80 mW cm^−2^. The fluorescence signals were captured with an EMCCD camera (Evolve delta, Photometrix) with appropriate filter sets (Chroma). DnaB loading was identified by colocalisation of DNA foci with DnaB_6_(red) foci to within 2 pixels (see Data analysis). In the loaded-DnaB stability experiments, the injection of the loading solution was followed by injection of single-molecule loading buffer at 100 μl min^−1^ for 1 min and then at 10 μl min^–1^ for 35 min. DNA was excited in a similar manner as the loading experiment; however, DnaB_6_(red) was excited in intervals of 400 ms once every 8 s to significantly extend the lifetime of the fluorophores to approximately 60 min (Supplementary Figure S4).

### Single-molecule rolling-circle replication assay

#### Preloaded conditions

This replication assay used the same microfluidic flow-cell devices described above as well as the same DnaB-loading step, followed by a replication step that was adapted from existing methods (29,31,38,42). In preloaded DnaB experiments, the replication solution contained 10 nM Pol III αϵθ core, 3.3 nM τ_3_δδ’ψχ clamp loader (i.e. Pol III* assembled *in situ* from clamp loader and cores in 4 μl for 90 s at 37°C), 30 nM β_2_ clamp, 75 nM DnaG and 20 nM SSB_4_ in single-molecule replication buffer (single-molecule loading buffer + 250 μM of each dNTP, 250 μM of each NTP, 1 mM UV-aged Trolox, 0.8% w/v glucose, 0.12 mg ml^–1^ glucose oxidase and 12 μg ml^–1^ catalase). Replication was initiated by injecting the replication solution into a flow chamber containing immobilised rolling-circle DNA templates at 100 μl min^−1^ for 1 min and then at 10 μl min^−1^ for 10 min. DNA and DnaB_6_(red) were visualised using the same conditions as the Single-molecule loading assay for a period of 10 min.

#### In-solution conditions

For the in-solution DnaB_6_(red) experiments, 2 nM DnaB_6_(red) was added to the replication solution. DnaC is not present here.

#### Chase conditions

For the WT DnaB_6_ chase experiments, 30 nM WT DnaB_6_ was added to the replication solution. The preloaded DnaB_6_(red) fluorescence lifetime was extended by exciting intermittently for 400 ms once every 800 ms. For the DnaB_6_(blue) chase experiments, 2 nM DnaB_6_(blue) was added to the replication solution. DnaB_6_(blue) was visualised by excitation with a 488-nm laser (Coherent, Sapphire 488-200 CW) at 4500 mW cm^−2^.

#### Fluorescence recovery after photobleaching (FRAP) conditions

For the DnaB_6_(red) FRAP experiments, 2 nM DnaB_6_(red) was added to the replication solution and after 2 min of imaging replication, a 647-nm FRAP pulse at 240 W cm^–2^ for 5 s was used to bleach all of the DnaB_6_(red) foci in the field of view. Recovery of any foci was recorded over the following 4 min.

### Stationary replisome association assay

The single-molecule replisome association assay was designed to detect the frequency with which free DnaB molecules interact with the replisome. This assay was identical to the DnaB_6_(blue) chase experiments, except for the replication/association step. In these experiments, dATP and dTTP were omitted. The remaining dCTP and dGTP are the next three nucleotides to be incorporated at the primer–template junction and thus keep the Pol III core bound in either the polymerization or proofreading mode and not synthesizing new DNA (Supplementary Figure S5). This technique has previously been applied to pre-assemble the whole replisome prior to replication (31,44,45), and to direct Pol III core towards the polymerisation or proofreading mode (46).

Therefore, in the whole replisome experiments, DnaB association was observed after injecting the association solution (replication solution without dATP or dTTP + 2 nM DnaB_6_(blue)) into a flow chamber containing immobilised rolling-circle DNA templates at 100 μl min^−1^ for 1 min and then at 10 μl min^−1^ for 10 min. As with the DnaB_6_(blue) chase experiments, this experiment was imaged by switching between DnaB_6_(red) and DnaB_6_(blue), and SYTOX Orange stained DNA was imaged once every 30 s for a total of 10 min. When indicated, replisome components were omitted to test their effect on the DnaB binding frequency.

### Data analysis

All analyses were carried out using ImageJ/Fiji (1.51e) and MATLAB 2016b, and in-house built plugins. Many of these processes are detailed elsewhere (41).

#### Quantification of degree of labelling

The numbers of fluorophores per labelled DnaB_6_(red) and DnaB_6_(blue) were quantified by immobilisation of DnaB_6_ from a 20 pM solution in replication buffer on the surface of a cleaned microscope coverslip. Imaging was done by exciting constantly at 2400 mW cm^–2^ for 5 min to allow photobleaching of the fluorophores. Raw movies were corrected for the electronic offset and excitation-beam profile. Single molecules of labelled DnaB_6_ were identified using our peak fitter tool and the photobleaching steps were fit using change-point analysis (Supplementary Figure S6A and D) (47–49). The histogram of steps per molecule was fit to a Poisson distribution (Supplementary Figure S6C and F). The degree of labelling was found to be 3–4 dyes (Poisson mean parameter, *λ* = 3.50; number of observations, *n* = 260) and 2–3 dyes (*λ* = 2.52; *n* = 181) per hexamer of DnaB_6_(red) and DnaB_6_(blue), respectively.

#### Determination of stoichiometry

The number of labelled DnaB_6_ molecules (during the loading step or at actively replicating replisomes) was calculated by dividing their initial intensities by the intensity of a single fluorophore and correcting for the pre-determined degree of labelling. The average intensity per fluorophore was quantified by detecting photobleaching steps in labelled DnaB_6_ non-specifically bound to the surface (Supplementary Figure S6B and E). The integrated intensity for every fluorescent DnaB in a field of view was calculated after applying a local background subtraction. The histograms obtained were fit with a Gaussian distribution function to give the average intensity.

#### Colocalisation analysis

Foci of two separate colours were classed as being colocalised if their centroid positions (determined using our peak fitter tool) fell within 2 pixels of each other. The chance of coincidental colocalisation (*C*) was calculated using Equation 1, where *A*_R_ is the focus area, *A*_FOV_ is the field of view area, and *n* is the number of foci.

(1) $$\begin{equation*}C\; = \frac{{{A_{\rm R}}}}{{{A_{{\rm FOV}}}}}\;\times n\end{equation*}$$

#### FRAP recovery analysis

To obtain the characteristic exchange time *τ* from the FRAP experiments, the intensity of DnaB was tracked as a function of time. The data were fit with a FRAP recovery function correcting for photobleaching (22,29,31,50) (Equation 2, where *a* is the amplitude of photobleaching, *τ_b_* is the photobleaching time, and *I*_0_ is the number of DnaB at the fork at steady state).

(2) $$\begin{equation*}I\; = a\times{{\rm e}^{ - \frac{t}{{{\tau _{\rm b}}}}}} + {I_0}\;\left( {1 - {{\rm e}^{ - \frac{t}{\tau }}}} \right)\end{equation*}$$

#### DnaB association frequency analysis

The characteristic timescales of the DnaB dynamics were extracted by tracking the fluorescence intensity of DnaB_6_(blue) over time. A threshold was determined for each intensity trajectory equivalent to the intensity of half a DnaB_6_(blue) molecule. The binding frequency was defined as the number of times per minute where the intensity exceeded the threshold.

## RESULTS

### Single-molecule visualisation of DnaB helicase dynamics

Previous studies have established single-molecule fluorescence methods to monitor reconstituted replisomes during *in vitro* DNA replication (28,29,31,32,44,45,51–54). Several of these studies also explore how functional *E. coli* replisomes can be sequentially assembled *in vitro* by introducing the appropriate proteins over time to immobilised DNA templates (29,31,50). We adapted these replisome assembly conditions to separate helicase loading and DNA replication into discrete steps and thus to examine DnaB activity in each.

To visualise single molecules of DNA-loaded DnaB, we used a mutant of DnaB_6_ (H201C) site-specifically labelled with a cysteine-reactive red fluorescent dye (Alexa Fluor 647). Note that wild-type DnaB contains no native cysteine residues, and His201 is positioned in a solvent-exposed loop near the C-terminal face of the helicase, remote from the sites of its interaction with DnaG (PDB ID: 2R6A) (55), the DnaC helicase loader and ssDNA (PDB ID: 6QEM) (4) (Supplementary Figure S2). Consistent with this location, neither the mutation nor subsequent labelling significantly affected the function of DnaB during leading-strand replication (Supplementary Figure S3). Nevertheless, we have not been able to exclude that interactions with other proteins (e.g. DnaA or Rep) that are not used in this work may be affected. It is also possible that the helicase activity of the mutant may be modestly affected by modulation of interactions with its excluded (leading) strand, as has been observed for a series of conserved basic residues on its surface (56); note that these residues are also structurally remote from H201, and interactions of DnaB with the excluded strand have not yet been characterised structurally.

**Figure 2.**
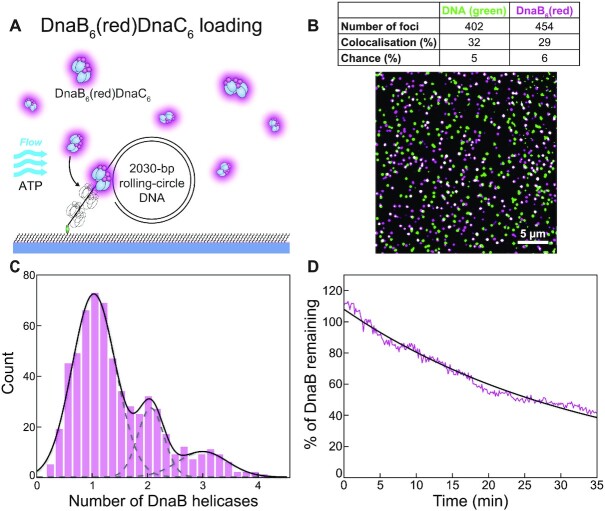
Visualisation of loaded DnaB helicases at the single-molecule level. (**A**) Illustration of the singlemolecule helicase-loading assay. DnaB_6_(red)DnaC_6_, a 2030 bp rolling-circle DNA template and the nucleotides required for loading are mixed and applied to a microfluidic flow channel. The 5′-biotinylated DNA couples to the streptavidin-functionalised surface and immobilises the complex. (**B**) Loaded DnaB helicases appear as colocalised foci (white) of DnaB_6_(red) and SYTOX Orange-stained DNA (green). The table indicates the number of foci, the degree of colocalisation and the degree of coincidental colocalisation by chance. (**C**) Distribution of DnaB_6_(red) stoichiometry loaded onto the 59-nt single-stranded DNA tail (*n* = 606). The black line represents the sum of three Gaussian distribution functions fit to the data. The dashed grey lines represent the individual Gaussian distributions. (**D**) The average binding lifetime of loaded DnaB_6_(red) molecules (magenta; *n* = 123). A single-exponential fit to the data (black) gives a binding lifetime of 34.4 ± 0.4 min. Photobleaching time is measured to be ∼60 min (Supplementary Figure S4) and therefore does not significantly impact on the observed kinetics.

A 5′-biotinylated rolling-circle DNA template (2030 bp) with replication fork topology (43) was used for DnaB loading and subsequent replisome assembly (Supplementary Figure S5A). DnaB_6_(red) mixed with DnaC in the presence of ATP was added to the DNA template and then injected into a microfluidic flow-cell to immobilise the complex on a streptavidin-functionalised surface (Figure 2A). The DNA template stained with SYTOX Orange (green) and DnaB_6_(red) were both visualised by near-total internal reflection fluorescence (TIRF) imaging and loaded DnaB helicases identified by the colocalization of the corresponding foci (Figure 2B). Of the DNA template foci detected in one field of view, 32% (*n* = 128) colocalise with DnaB_6_(red) foci and thus represent successfully loaded DnaBC helicase–helicase loader complexes. This value is well above the degree of colocalisation expected by chance (5%; see Methods).

To calculate the number of DnaB helicases loaded on DNA templates, we quantified the intensities of colocalised DnaB_6_(red) foci and divided them by the calibrated average intensity for a single DnaB molecule (Supplementary Figure S6B). The resulting distribution shows three distinct populations corresponding to 1.0 ± 0.1, 2.0 ± 0.1 and 2.9 ± 0.1 loaded helicases (mean ± S.E.M., *n* = 606) with decreasing occurrence, respectively (Figure 2C). The 59-nt single-stranded tail of the DNA template (Supplementary Figure S5A) could plausibly accommodate up to two DnaBC complexes with an estimated footprint of ∼30 nt (4) or three DnaB after DnaC dissociation with a footprint of 18–20 nt per helicase hexamer (57). It is also possible that one molecule of DnaB could occupy the 25-nt gap on the leading strand. The shape of the distribution (Figure 2C) implies that the probability of loading another helicase decreases as available ssDNA space decreases.

We assessed the stability of DnaB helicases loaded at the fork by flowing out excess DnaB_6_(red) (and DnaC) and measuring the binding lifetime of loaded DnaB_6_(red) (Figure 2D). The average intensity over time of *n* = 123 DnaB_6_(red) molecules was fit to a single exponential decay, yielding a mean lifetime of 34.4 ± 0.4 min (mean ± S.E.M.). The photobleaching of DnaB_6_(red) was greatly extended by imaging intermittently for 400 ms once every 8 s. To determine the photobleaching lifetime under these conditions, we extrapolated several known values for other conditions to determine a value of ∼60 min (Supplementary Figure S4). It is unlikely that this degree of photobleaching interferes with the measurement of the DnaB binding lifetime. Even taking the impact of photobleaching into account, our data suggest that once loaded, DnaB remains stably bound at a fork for ∼30 min.

**Figure 3.**
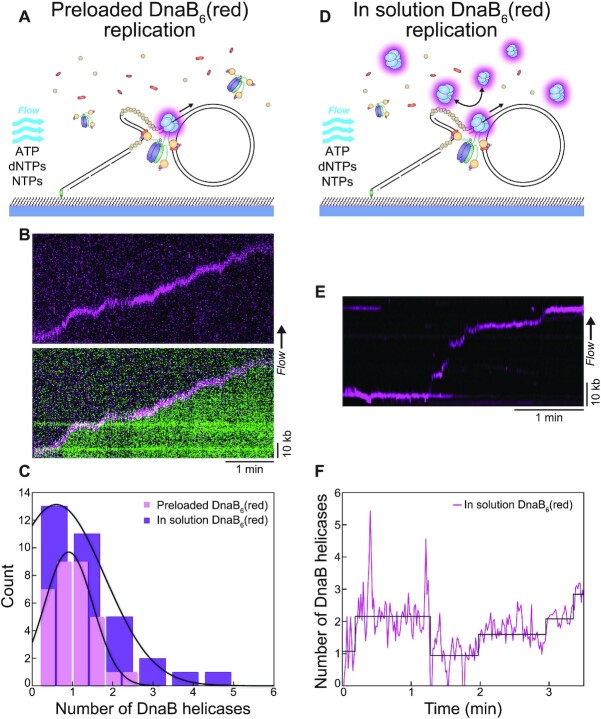
More than one DnaB helicase are frequently present at the replication fork. (**A**) Illustration of the rolling-circle replication assay with DnaB_6_(red) preloaded on the DNA prior to introducing the replication solution. Replication is monitored in real time by flow stretching the replicating DNA products by hydrodynamic force. (**B**) (Top) Representative kymograph of preloaded DnaB_6_(red) moving with the fork during rolling-circle replication. (Bottom) Overlay of the DnaB_6_(red) and SYTOX Orange-stained DNA (green) kymographs shows the helicase molecule moving with the replication fork at the tip of the DNA product. (**C**) Distributions of DnaB helicase stoichiometry at the fork in the absence of DnaB_6_(red) in solution (magenta; *n* = 32) and in the presence of DnaB_6_(red) in solution (purple; *n* = 33). The black lines represent Gaussian fits to the data. (**D**) Illustration of the in-solution assay, where DnaB_6_(red) is preloaded and also included at 2 nM in the replication solution. (**E**) Representative kymograph showing the DnaB_6_(red) signal at the fork when DnaB_6_(red) is present in solution. (**F**) Number of DnaB_6_(red) as a function of time for the kymograph in (E) showing the fluctuation in DnaB helicase stoichiometry during the course of replication, where steps are detected by change-point analysis (47–49).

After our observation of the number of helicases loaded at the fork, we set out to determine the number associated with progressing replisomes. Similar to the helicase loading experiments, we pre-loaded DnaB_6_(red) on the rolling-circle DNA template and immobilised the complex on the surface. Then, we flow out excess DnaB_6_(red) (and DnaC) and flow in replication solution (Pol III*, β_2_ clamp, DnaG primase, SSB, dNTPs, NTPs) to initiate the replication reaction (Figure 3A). Rolling-circle replication products could be observed within tens of seconds as they were stretched out by hydrodynamic force, where the start of the kymograph indicates the point of buffer injection (Figure 3B, Supplementary Figure S7A). Intermittent imaging of SYTOX Orange stained DNA (green) and DnaB_6_(red) identified DnaB helicases present only at the replication fork of DNA products (Figure 3B). Stoichiometric quantification of these DnaB_6_(red) foci produced a distribution centred about 0.9 ± 0.1 helicases (mean ± S.E.M., *n* = 32) (Figure 3C). Therefore, these results confirmed that of the multiple DnaB helicases stably loaded onto ssDNA, only one is converted into an active replisome.

When we repeat this rolling-circle replication experiment, but now with 2 nM DnaB_6_(red) present in the replication solution, we observe a different outcome (Figure 3D). DnaB_6_(red) foci were found at the fork (Figure 3E) but also occasionally left behind the progressing replisome (Supplementary Figure S7B). We considered the possibility that DnaB_6_(red) might be binding to incomplete Okazaki fragments with ssDNA gaps under these conditions where excess Pol III holoenzyme and SSB, but not DnaC, are present in solution. Although DnaB is known to associate with naked ssDNA (57,58), its loading onto SSB-coated ssDNA generally requires specialized DNA structures such as forks, DnaC and other (replication restart) proteins (58–60). Although ssDNA gaps on the lagging strand can be observed when experiments are set up specifically to detect them (61,62) and may be produced under our conditions due to premature release of the lagging strand polymerase, we have shown that these gaps are subsequently filled by the additional free Pol III holoenzyme from solution (29). We conclude therefore that the labelled DnaB is most likely bound *via* the τ subunit (50) to the Pol III holoenzyme left behind at Okazaki fragment junctions under these conditions where Okazaki fragment processing enzymes (DNA Pol I and ligase) are absent (29).

Analysis of the stoichiometry of DnaB_6_(red) localised at the fork results in a distribution centred at 0.6 ± 0.3 helicases (mean ± S.E.M., *n* = 33). Examining individual intensity traces of DnaB_6_(red) shows that intermittently, there is an increase in fluorescence equivalent to another helicase molecule localised to the replisome (Figure 3F). The physical divergence of these two helicase signals (Figure 3E) suggests one supports rolling circle replication, while the other does not. The presence of more than one helicase raises two questions: Is the replisomal DnaB helicase exchanging with other helicases in the surrounding solution? Or conversely, are extra helicases contributing to the replisome *via* a secondary mechanism?

### DnaB helicase is a stable anchor within the replisome

There is no evidence in the literature to suggest that DnaB stochastically exchanges during DNA replication in the same manner shown for Pol III* and SSB (22,29,31). We detect, however, recurrent dynamics in the DnaB_6_(red) signal, indicating there is often more than one helicase at the replication fork (Figure 3E and F), and thus the potential for helicase exchange. Similar to other single-molecule studies of exchange, we applied fluorescence recovery after photobleaching (FRAP) to our replication assay with DnaB_6_(red) present in solution. Recovery events were detected (Supplementary Figure S8A and B) which indicates DnaB_6_(red) molecules from solution associate with the fork during replication. Summing multiple single-molecule recovery curves suggests a recovery on the timescale of several minutes (Supplementary Figure S8C). While suggestive of dynamic exchange, the apparent on/off events in the fluorescence signal creates noise in the FRAP recovery curves, making the process challenging to study.

**Figure 4.**
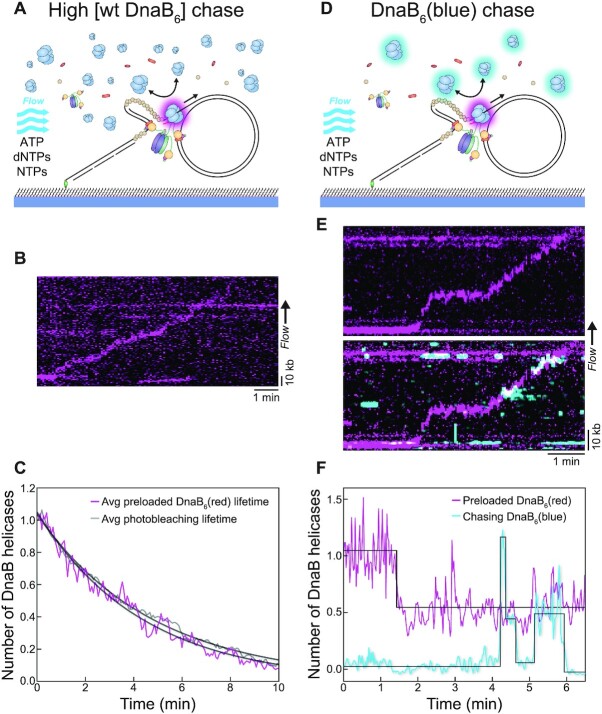
DnaB helicases are both stable and dynamic during replication. (**A**) Illustration of the WT DnaB chase assay, where preloaded DnaB_6_(red) was ‘chased’ with a relatively high concentration of WT DnaB (30 nM) in the replication solution. Like the standard rolling-circle assay, DNA products are stretched out by hydrodynamic force. (**B**) Representative kymograph of DnaB_6_(red) moving with the fork during rolling-circle replication in the WT DnaB chase assay. (**C**) The average intensity over time from replicating DnaB_6_(red) molecules in the WT DnaB chase assay (magenta; *n* = 29), compared to the photobleaching lifetime of DnaB_6_(red) (grey; *n* = 667). The curve from each condition is fit with a single-exponential decay to provide the characteristic lifetime. (**D**) Illustration of the DnaB_6_(blue) chase assay, where preloaded DnaB_6_(red) is ‘chased’ with DnaB_6_(blue) (2 nM) in the replication solution. Again, the DNA products are stretched out by hydrodynamic force. (**E**) (Top) Representative kymographs of DnaB_6_(red) moving with the fork during rolling-circle replication in the DnaB_6_(blue) chase assay. (Bottom) The DnaB_6_(blue) kymograph from the same replication event shows the frequent association of additional helicases with the replication fork. (**F**) The stoichiometry over time from both the DnaB_6_(red) and DnaB_6_(blue) signal corresponding to the kymograph in (E), where steps are detected by change-point analysis (47–49).

To further test the helicase exchange hypothesis, we developed a chase exchange assay as an alternative approach to address exchange during DNA replication. First, we initiated rolling-circle replication with pre-loaded DnaB_6_(red) and then chased with a physiological concentration (30 nM) of dark, WT DnaB_6_ in the replication solution (Figure 4A). We hypothesised that if fluorescent DnaB exchanges with unlabelled DnaB from solution, we should see a disappearance of fluorescence at the fork. However, we found replisomal DnaB_6_(red) molecules to be unaffected by the excess unlabelled, WT DnaB_6_ during replication (Figure 4B, Supplementary Figure S7C). Analysing the fluorescence of these replicating DnaB_6_(red) foci identified that the average lifetime of 4.6 ± 0.1 min (mean ± S.E.M., *n* = 29) is very close to that of the photobleaching lifetime of 4.8 ± 0.1 min (mean ± S.E.M., *n* = 667) (Figure 4C). The inability of the WT DnaB_6_ to perturb replisomal DnaB_6_(red) is compelling evidence that the helicase does not undergo exchange during DNA replication. Instead, these results suggest a single DnaB helicase persists for the entirety of the replication cycle, acting as a stable anchor for the other replisome components.

To further investigate the extra DnaB_6_(red) foci identified at the replication fork, we applied a similar technique to the dark WT DnaB_6_ chase experiment. In this experiment, DnaB_6_(red) was pre-loaded onto the rolling-circle DNA template, but then chased with DnaB_6_(blue) in the replication solution (Figure 4D). Imaging both colours of labelled DnaB during replication showed DnaB_6_(red) foci at the replication fork in parallel to our previous observations, but also DnaB_6_(blue) molecules binding intermittently at the fork (Figure 4E, Supplementary Figure S7D). The appearance of DnaB_6_(blue) fluorescence did not correlate with the disappearance of DnaB_6_(red) signal (Figure 4F), which further solidifies the absence of helicase exchange. The dynamic association of DnaB_6_(blue) with the replication fork suggests extra DnaB helicases can somehow transiently interact with the replisome.

### Additional helicases dynamically interact with the replisome through the τ subunit of the CLC

**Figure 5.**
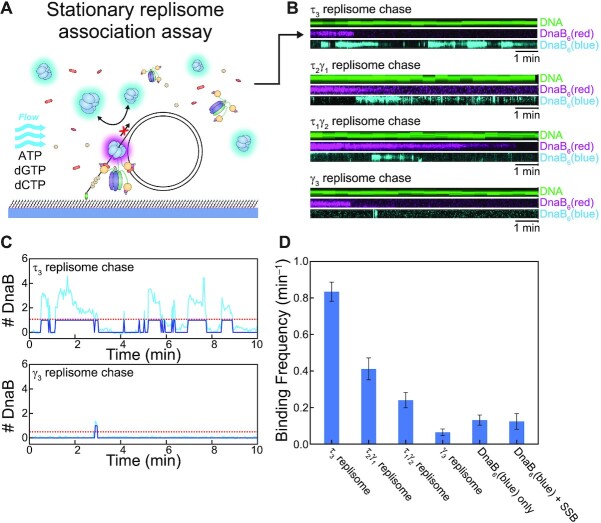
Additional helicases are able to interact with the replisome through the τ subunit of the clamploader complex. (**A**) Illustration of the stationary replisome-association assay. The replisome (including DnaB_6_(blue)) is assembled during the ‘association’ phase after preloading DnaB_6_(red) onto DNA. Including only dGTP and dCTP in this reaction permits the replisome to assemble but precludes net DNA synthesis. (**B**) Example kymographs from different experiments where the CLC has a varying composition of τ subunits. Comparing the DnaB_6_(blue) kymographs shows how changing the reaction composition affects the frequency at which free DnaB_6_(blue) binds to assembled replisomes. More example kymographs can be found in the Supplementary Figure S9. (**C**) The detection of DnaB_6_(blue) binding from the example kymographs from the τ_3_ replisome chase and γ_3_ replisome chase experiments in (B). A binding event is recorded (dark blue) when the intensity of the DnaB_6_(blue) signal (light blue) passes the threshold level (red). (**D**) Comparison of the binding frequency of DnaB_6_(blue) from different experiments: τ_3_ replisome, *n* = 123; τ_1_γ_2_ replisome, *n* = 70; γ_3_ replisome, *n* = 43; DnaB_6_(blue) only, *n* = 74; DnaB_6_(blue) and SSB only, *n* = 36. Tabulation of these values can be found in Supplementary Figure S11.

Our observation of the presence of extra helicases at the replication fork has not been reported before. Our chase replication experiments establish that any additional helicases do not interfere with the unwinding function of the main replisomal DnaB helicase. To understand the nature of the interaction of the extra DnaB helicases, we restructured our replication assay into a stationary replisome association assay. In this assay we utilise our available fluorescent tools to assess association of fluorescent DnaB with a stationary replisome to achieve much higher throughput of data. This stationary replisome association assay builds on observations of single-molecule DNA replication as carried out by replisomes completely pre-assembled prior to replication (31,44,45). First, the loaded DnaB_6_(red)DnaC_6_–DNA complex (identical to the DnaB-loading experiments in Figure 2A) is immobilised on the surface of a flow-cell. Then, an association solution consisting of the remaining replisome components, DnaB_6_(blue), ATP, dCTP and dGTP is injected (Figure 5A). Including only two of the four nucleotides allows for proper binding of the polymerases within the replisome but prevents them from replicating the whole template (Supplementary Figure S5B). When the polymerase core is blocked from synthesis, it is expected to enter a stalled state which still retains a strong affinity for DNA (46), and thus the entire replisome can be assembled but remains stationary. Herein lies the effectiveness of this assay, as we can identify assembled replisomes and then automate the acquisition and analysis of DnaB_6_(blue)-replisome interactions.

We identified stationary, assembled replisomes based on the colocalisation of DnaB_6_(red) and SYTOX Orange-stained DNA in the same manner as the DnaB loading experiments (Supplementary Figure S9A). When the immobilised DnaB_6_(red)DnaC_6_-DNA complexes are exposed to the association solution with all the replisome components, we see dynamic binding of DnaB_6_(blue) molecules (Figure 5B). These dynamics are consistent with the association events detected at the replication fork during the DnaB_6_(blue) chase assay (Figure 4E). When we analyse all the intensities observed throughout the experiment, we see that, on average, DnaB_6_(blue) is bound 43% of the time (Supplementary Figure S10A). In contrast, when we omit all other replisome components from the association solution, we only see DnaB_6_(blue) bound 12% of the time (Supplementary Figure S10B). To extract the characteristic frequency of the binding dynamics, we applied thresholding analysis to the DnaB_6_(blue) signal to automate the detection of binding events (Figure 5C). We found that the binding frequency of DnaB_6_(blue) varied both within and between experiments depending on several factors. For the condition where all replisome components are present (Figure 5D), we determined a binding frequency of 0.84 ± 0.05 min^–1^ (mean ± S.E.M., *n* = 123). We are able to compare this measurement to other DnaB_6_(blue) binding populations within the same experiment. When we measure the DnaB_6_(blue) dynamics on DNA templates that lack a loaded DnaB_6_(red), the frequency was slightly lower (0.69 ± 0.01 min^–1^, *n* = 1651; Supplementary Figure S11). The dynamics on DnaB_6_(red) foci not bound to DNA are less frequent (0.35 ± 0.02 min^–1^, *n* = 421; Supplementary Figure S11). Together these observations show that the additional DnaB interactions occur more frequently at replisomes containing all components. Furthermore, when we modify the experimental condition to include only DnaB_6_(blue) in the association solution, we observe significantly less DnaB_6_(blue) binding at colocalised DnaB_6_(red)–DNA sites (0.13 ± 0.02 min^–1^, *n* = 74) (Figure 5D). The lack of dynamics in the absence of other replisomal components indicates that the additional DnaB binds to the replisome through an interaction with another replisomal protein.

To identify this factor, we repeated the experiment with the omission of specific proteins and found that when the CLC lacks the τ subunit (γ_3_δδ’ψχ), significantly less frequent DnaB_6_(blue) binding is detected. Under these conditions, the binding frequency of DnaB_6_(blue) at DnaB_6_(red)–DNA sites, 0.07 ± 0.02 min^–1^ (*n* = 43) is not significantly different from that observed with DnaB_6_(blue) only (above) or with only SSB and DnaB_6_(blue) (0.12 ± 0.04 min^–1^, *n* = 36) (Figure 5D). Part of the DnaB_6_(blue) binding dynamics can be recovered proportionally by the presence of a CLC that includes either one (τ_1_γ_2_δδ’ψχ; 0.24 ± 0.04 min^–1^, *n* = 70) or two τ subunits (τ_2_γ_1_δδ’ψχ; 0.41 ± 0.06 min^–1^, *n* = 26) (Figure 5D). Taken together, these results implicate the τ subunit of Pol III holoenzyme as the factor responsible for recruiting extra DnaB helicases to the replisome. DnaB has a known affinity for τ (16,50), yet it was always believed that this interaction was reserved for maintaining the physical connection between the Pol III HE and the replisomal helicase.

## DISCUSSION

We report here the use of single-molecule fluorescence imaging to visualise single molecules of the DnaB helicase and study its dynamics. We used a collection of functional assays to examine the stability of the helicase during the discrete stages of helicase loading and DNA replication.

Firstly, our quantification of labelled DnaB stoichiometry showed that during loading, multiple DnaB molecules can be deposited onto the exposed single-stranded region of our DNA template. In contrast, quantitative studies of replication initiation identified that each strand of the *E. coli* origin of replication, *oriC* is bound by a single DnaBC complex (63,64). Our loading method, although effective for our assay, does not reproduce the physiological conditions of initiation at *oriC*. We used a pre-made fork template with an exposed 5′ end in contrast to the topologically closed DNA bubble that occurs at *oriC*. We also do not include the DnaA initiator protein, which normally recruits DnaB to the origin through interactions with both DnaB and DnaC (64–69). Our results demonstrate that multiple helicases will associate with ssDNA if there is space available. Therefore, we know DnaBC is capable of engaging ssDNA by itself, but special mechanisms are in play at *oriC* involving DnaA to load two and only two helicases onto opposite strands.

We find that, once loaded, DnaB remains stably associated with the forked ssDNA for ∼30 min. This lifetime of DnaB during loading is comparable to other observations of DnaB being bound to ssDNA for times between 5 and 30 min (22–26). Our results are further evidence that once DnaB associates with DNA, it forms a highly stable nucleoprotein complex. The DnaB helicase is also expected to be stably integrated as part of the active replisome. Other studies have inferred the stability of replisomal DnaB based on *in vivo* lifetime measurements (22), and the stability of the whole replisome during normal DNA replication (70) or once challenged (23–26). It is nevertheless possible that hidden amongst these indirect measurements of stability, DnaB has the capacity to exchange during replication. There are an increasing number of studies demonstrating that, at cellular protein concentrations, the bacterial replisome does not have a static composition, but rather exchanges its components frequently (22,27–32,71). Therefore, we designed three single-molecule replication assays to directly detect DnaB exchange. Each of these showed the absence of exchange on the minutes timescale—instead the replisomal helicase was maintained for the entirety of replication. This result implicates DnaB as the stability factor of the replisome on which other components can dynamically interchange, and agrees with the regulatory mechanism suggested by Monachino *et al.* (50). Our direct observations of DnaB stability indicates its function as the processivity factor of the replisome. The replisomal helicase not only forms the stable anchor on which exchange occurs, but also acts as the central platform for the coordination of the main events of replication.

Despite the lack of DnaB exchange in the replisome, we do detect recurrent dynamics in the DnaB signals. These dynamics signify the frequent and transient association of extra helicases with the replication fork. From our chase experiments, we demonstrate that these extra helicases are indeed not stably incorporated, and do not interfere with the replisomal DnaB. Two other single-molecule studies of replisome stoichiometries in live cells find populations centred about one or two helicases (20,21). Both studies explain the two-helicase population as the result of two replisomes bidirectionally replicating the chromosome contained within a single diffraction-limited spot. The width of the stoichiometry distributions in both studies, however, could hide populations in which up to three or four hexamers are bound momentarily within these diffraction-limited spots. These observations are consistent with our finding of extra DnaB transiently interacting with the replisome.

It is unclear if additional helicases play a functional role in the replisome. Our observation of successful replication with and without extra helicases suggests that these DnaB molecules do not contribute to unwinding of dsDNA. Furthermore, as we have demonstrated the high degree of stability of the helicase, the benefit of having additional DnaB in the replisome is not readily apparent. One possible implication relates to cellular DNA lesions (60), where re-loading of DnaB after lesion-induced stalling could be helped by readily available extra helicases. Through quantification of the frequency of DnaB associating with stationary replisomes, we determined that additional helicases interact with the replisome through the τ subunit(s) of the clamp-loader complex. With increasing number of τ subunits in the CLC, we see a proportionate increase in the binding frequencies of additional helicases. This dependency shows that the local concentration of τ subunits dictates the access of free helicases from solution. The mode of this interaction is likely the same as the previously identified weak interaction of domain IV of τ and DnaB (50). It remains difficult to pinpoint the exact number of replisomal helicase connections and if such factors are static or dynamic throughout the Okazaki fragment cycling of the replisome.

In conclusion, we applied single-molecule imaging tools to an *in vitro*-reconstituted *E. coli* replication reaction to demonstrate a high stability of integration of the replicative DnaB helicase at replication forks. We argue that DnaB is the stable anchor within the replisome that plays a critical role in replisome processivity and thus integrity. Having established a fluorescence-based assay for visualisation of DnaB at the single-molecule level, future experimentation should focus on the dynamics of replication initiation as well as alternative pathways sampling by DnaB and the CLC during the cycling of Okazaki fragments. Such studies would help us understand how the DnaB helicase, and by extension the whole replisome, coordinates with other proteins to respond to challenges to replication integrity.

## DATA AVAILABILITY

Home-built ImageJ plugins have been deposited on the Github repository for Single-molecule/Image analysis tools (https://github.com/SingleMolecule).

## Supplementary Material

gkab493_Supplemental_File

## References

[1] Lewis J.S. , Jergic S., Dixon N.E. The *E. coli* DNA replication fork. Enzymes. 2016; 39:31–88. 27241927 10.1016/bs.enz.2016.04.001

[2] Leipe D.D. , Aravind L., Grishin N.V., Koonin E.V. The bacterial replicative helicase DnaB evolved from a RecA duplication. Genome Res. 2000; 10:5–16. 10645945

[3] Chodavarapu S. , Kaguni J.M. Replication initiation in bacteria. Enzymes. 2016; 39:1–30. 27241926 10.1016/bs.enz.2016.03.001 PMC5551690

[4] Chodavarapu S. , Jones A.D., Feig M., Kaguni J.M. DnaC traps DnaB as an open ring and remodels the domain that binds primase. Nucleic Acids Res. 2016; 44:210–220. 26420830 10.1093/nar/gkv961 PMC4705694

[5] Felczak M.M. , Chodavarapu S., Kaguni J.M. DnaC, the indispensable companion of DnaB helicase, controls the accessibility of DnaB helicase by primase. J. Biol. Chem. 2017; 292:20871–20882. 29070678 10.1074/jbc.M117.807644 PMC5743064

[6] Arias-Palomo E. , Puri N., O'Shea Murray V.L., Yan Q., Berger J.M. Physical basis for the loading of a bacterial replicative helicase onto DNA. Mol. Cell. 2019; 74:173–184. 30797687 10.1016/j.molcel.2019.01.023 PMC6450724

[7] Makowska-Grzyska M. , Kaguni J.M. Primase directs the release of DnaC from DnaB. Mol. Cell. 2010; 37:90–101. 20129058 10.1016/j.molcel.2009.12.031 PMC2819048

[8] Lu Y.B. , Ratnakar P.V., Mohanty B.K., Bastia D. Direct physical interaction between DnaG primase and DnaB helicase of *Escherichia coli* is necessary for optimal synthesis of primer RNA. Proc. Natl. Acad. Sci. U.S.A. 1996; 93:12902–12907. 8917517 10.1073/pnas.93.23.12902 PMC24018

[9] Corn J.E. , Pease P.J., Hura G.L., Berger J.M. Crosstalk between primase subunits can act to regulate primer synthesis in trans. Mol. Cell. 2005; 20:391–401. 16285921 10.1016/j.molcel.2005.09.004

[10] Kelman Z. , O’Donnell M. DNA polymerase III holoenzyme: structure and function of a chromosomal replicating machine. Annu. Rev. Biochem. 1995; 64:171–200. 7574479 10.1146/annurev.bi.64.070195.001131

[11] Leu F.P. , Hingorani M.M., Turner J., O’Donnell M. The δ subunit of DNA polymerase III holoenzyme serves as a sliding clamp unloader in *Escherichia coli*. J. Biol. Chem. 2000; 275:34609–34618. 10924523 10.1074/jbc.M005495200

[12] Dohrmann P.R. , McHenry C.S. A bipartite polymerase–processivity factor interaction: only the internal β binding site of the α subunit is required for processive replication by the DNA polymerase III holoenzyme. J. Mol. Biol. 2005; 350:228–239. 15923012 10.1016/j.jmb.2005.04.065

[13] Jergic S. , Horan N.P., Elshenawy M.M., Mason C.E., Urathamakul T., Ozawa K., Robinson A., Goudsmits J.M.H., Wang Y., Pan X. et al. A direct proofreader-clamp interaction stabilizes the Pol III replicase in the polymerization mode. EMBO J. 2013; 32:1322–1333. 23435564 10.1038/emboj.2012.347 PMC3642676

[14] Gao D. , McHenry C.S. τ binds and organizes *Escherichia coli* replication proteins through distinct domains. Partial proteolysis of terminally tagged τ to determine candidate domains and to assign domain V as the α binding domain. J. Biol. Chem. 2001; 276:4433–4440. 11078743 10.1074/jbc.M009828200

[15] Gao D. , McHenry C.S. τ binds and organizes *Escherichia coli* replication proteins through distinct domains. Domain IV, located within the unique C terminus of τ, binds the replication fork helicase, DnaB. J. Biol. Chem. 2001; 276:4441–4446. 11078744 10.1074/jbc.M009830200

[16] Jergic S. , Ozawa K., Williams N.K., Su X.-C., Scott D.D., Hamdan S.M., Crowther J.A., Otting G., Dixon N.E. The unstructed C-terminus of the τ subunit of *Escherichia coli* DNA polymerase III holoenzyme is the site of interaction with the α subunit. Nucleic Acids Res. 2007; 35:2813–2824. 17355988 10.1093/nar/gkm079 PMC1888804

[17] Drake J.W. Comparative rates of spontaneous mutation. Nature. 1969; 221:1132. 4378427 10.1038/2211132a0

[18] Chandler M. , Bird R.E., Caro L. The replication time of the *Escherichia coli* K12 chromosome as a function of cell doubling time. J. Mol. Biol. 1975; 94:127–132. 1095767 10.1016/0022-2836(75)90410-6

[19] Perera H.M. , Behrmann M.S., Hoang J.M., Griffin W.C., Trakselis M.A. Contacts and context that regulate DNA helicase unwinding and replisome progression. Enzymes. 2019; 45:183–223. 31627877 10.1016/bs.enz.2019.08.001

[20] Reyes-Lamothe R. , Sherratt D.J., Leake M.C. Stoichiometry and architecture of active DNA replication machinery in *Escherichia coli*. Science. 2010; 328:498–501. 20413500 10.1126/science.1185757 PMC2859602

[21] Mangiameli S.M. , Merrikh C.N., Wiggins P.A., Merrikh H. Transcription leads to pervasive replisome instability in bacteria. eLife. 2017; 6:e19848. 28092263 10.7554/eLife.19848 PMC5305214

[22] Beattie T.R. , Kapadia N., Nicolas E., Uphoff S., Wollman A.J., Leake M.C., Reyes-Lamothe R. Frequent exchange of the DNA polymerase during bacterial chromosome replication. eLife. 2017; 6:e21763. 28362256 10.7554/eLife.21763 PMC5403216

[23] Maisnier-Patin S. , Nordström K., Dasgupta S. Replication arrests during a single round of replication of the *Escherichia coli* chromosome in the absence of DnaC activity. Mol. Microbiol. 2001; 42:1371–1382. 11886566 10.1046/j.1365-2958.2001.02718.x

[24] Labib K. , Hodgson B. Replication fork barriers: pausing for a break or stalling for time. EMBO Rep. 2007; 8:346–353. 17401409 10.1038/sj.embor.7400940 PMC1852754

[25] Pomerantz R.T. , O’Donnell M. Direct restart of a replication fork stalled by a head-on RNA polymerase. Science. 2010; 327:590–592. 20110508 10.1126/science.1179595 PMC2861996

[26] Mettrick K.A. , Grainge I. Stability of blocked replication forks *in vivo*. Nucleic Acids Res. 2016; 44:657–668. 26490956 10.1093/nar/gkv1079 PMC4737137

[27] Loparo J.J. , Kulczyk A.W., Richardson C.C., van Oijen A.M. Simultaneous single-molecule measurements of phage T7 replisome composition and function reveal the mechanism of polymerase exchange. Proc. Natl. Acad. Sci. U.S.A. 2011; 108:3584–3589. 21245349 10.1073/pnas.1018824108 PMC3048139

[28] Geertsema H.J. , Kulczyk A.W., Richardson C.C., van Oijen A.M. Single-molecule studies of polymerase dynamics and stoichiometry at the bacteriophage T7 replication machinery. Proc. Natl. Acad. Sci. U.S.A. 2014; 111:4073–4078. 24591606 10.1073/pnas.1402010111 PMC3964090

[29] Lewis J.S. , Spenkelink L.M., Jergic S., Wood E.A., Monachino E., Horan N.P., Duderstadt K.E., Cox M.M., Robinson A., Dixon N.E. et al. Single-molecule visualization of fast polymerase turnover in the bacterial replisome. eLife. 2017; 6:e23932. 28432790 10.7554/eLife.23932 PMC5419744

[30] Li Y. , Chen Z., Matthews L.A., Simmons L.A., Biteen J.S. Dynamic exchange of two essential DNA polymerases during replication and after fork arrest. Biophys. J. 2019; 116:684–693. 30686488 10.1016/j.bpj.2019.01.008 PMC6382952

[31] Spenkelink L.M. , Lewis J.S., Jergic S., Xu Z.-Q., Robinson A., Dixon N.E., van Oijen A.M. Recycling of single-stranded DNA-binding protein by the bacterial replisome. Nucleic Acids Res. 2019; 47:4111–4123. 30767010 10.1093/nar/gkz090 PMC6486552

[32] Lewis J.S. , Spenkelink L.M., Schauer G.D., Yurieva O., Mueller S.H., Natarajan V., Kaur G., Maher C., Kay C., O’Donnell M.E. et al. Tunability of DNA polymerase stability during eukaryotic DNA replication. Mol. Cell. 2020; 77:17–25. 31704183 10.1016/j.molcel.2019.10.005 PMC6943181

[33] Scherr M.J. , Safaric B., Duderstadt K.E. Noise in the machine: alternative pathway sampling is the rule during DNA replication. Bioessays. 2018; 40:1700159. 10.1002/bies.201700159 29282758

[34] Mueller S.H. , Spenkelink L.M., van Oijen A.M. When proteins play tag: the dynamic nature of the replisome. Biophys. Rev. 2019; 11:641–651. 10.1007/s12551-019-00569-4 PMC6682189 31273608

[35] Oakley A.J. , Prosselkov P., Wijffels G., Beck J.L., Wilce M.C.J., Dixon N.E. Flexibility revealed by the 1.85 Å crystal structure of the β sliding-clamp subunit of *Escherichia coli* DNA polymerase III. Acta Crystallogr. D. 2003; 59:1192–1199. 12832762 10.1107/s0907444903009958

[36] Mason C.E. , Jergic S., Lo A.T.Y., Wang Y., Dixon N.E., Beck J.L. *Escherichia coli* single-stranded DNA-binding protein: nanoESI-MS studies of salt-modulated subunit exchange and DNA binding transactions. J. Am. Soc. Mass Spectrom. 2013; 24:274–285. 23283730 10.1007/s13361-012-0552-2

[37] Stamford N.P.J. , Lilley P.E., Dixon N.E. Enriched sources of *Escherichia colil* replication proteins. The dnaG primase is a zinc metalloprotein. Biochim. Biophys. Acta. 1992; 1132:17–25. 1511009 10.1016/0167-4781(92)90047-4

[38] Tanner N.A. , Hamdan S.M., Jergic S., Loscha K.V., Schaeffer P.M., Dixon N.E., van Oijen A.M. Single-molecule studies of fork dynamics in *Escherichia coli* DNA replication. Nat. Struct. Mol. Biol. 2008; 15:170–176. 18223657 10.1038/nsmb.1381 PMC2651573

[39] Elvin C.M. , Thompson P.R., Argall M.E., Hendry P., Stamford N.P.J., Lilley P.E., Dixon N.E. Modified bacteriophage lambda promoter vectors for overproduction of proteins in *Escherichia coli*. Gene. 1990; 87:123–126. 2139621 10.1016/0378-1119(90)90503-j

[40] Kim Y. , Ho S.O., Gassman N.R., Korlann Y., Landorf E.V., Collart F.R., Weiss S. Efficient site-specific labeling of proteins via cysteines. Bioconj. Chem. 2008; 19:786–791. 10.1021/bc7002499 PMC3086356 18275130

[41] Geertsema H.J. , Duderstadt K.E., van Oijen A.M. Single-molecule observation of prokaryotic DNA replication. Methods Mol. Biol. 2015; 1300:219–238. 25916715 10.1007/978-1-4939-2596-4_14

[42] Spinks R.R. , Spenkelink L.M., van Oijen A.M. Single-molecule fluorescence methods to study protein exchange kinetics in supramolecular complexes. Methods Mol. Biol. 2021; 2281:49–65. 33847951 10.1007/978-1-0716-1290-3_3

[43] Monachino E. , Ghodke H., Spinks R.R., Hoatson B.S., Jergic S., Xu Z.-Q., Dixon N.E., van Oijen A.M. Design of DNA rolling-circle templates with controlled fork topology to study mechanisms of DNA replication. Anal. Biochem. 2018; 557:42–45. 30016625 10.1016/j.ab.2018.07.008

[44] Yao N.Y. , Georgescu R.E., Finkelstein J., O’Donnell M.E. Single-molecule analysis reveals that the lagging strand increases replisome processivity but slows replication fork progression. Proc. Natl. Acad. Sci. U.S.A. 2009; 106:13236–13241. 19666586 10.1073/pnas.0906157106 PMC2726342

[45] Tanner N.A. , Tolun G., Loparo J.J., Jergic S., Griffith J.D., Dixon N.E., van Oijen A.M. *E. coli* DNA replication in the absence of free β clamps. EMBO J. 2011; 30:1830–1840. 21441898 10.1038/emboj.2011.84 PMC3101994

[46] Park J. , Jergic S., Jeon Y., Cho W.K., Lee R., Dixon N.E., Lee J.-B. Dynamics of proofreading by the *E. coli* Pol III replicase. Cell Chem. Biol. 2018; 25:57–66. 29104063 10.1016/j.chembiol.2017.09.008

[47] Watkins L.P. , Yang H. Detection of intensity change points in time-resolved single-molecule measurements. J. Phys. Chem. B. 2005; 109:617–628. 16851054 10.1021/jp0467548

[48] Duderstadt K.E. , Geertsema H.J., Stratmann S.A., Punter C.M., Kulczyk A.W., Richardson C.C., van Oijen A.M. Simultaneous real-time imaging of leading and lagging strand synthesis reveals the coordination dynamics of single replisomes. Mol. Cell. 2016; 64:1035–1047. 27889453 10.1016/j.molcel.2016.10.028

[49] Hill F.R. , van Oijen A.M., Duderstadt K.E. Detection of kinetic change points in piece-wise linear single molecule motion. J. Chem. Phys. 2018; 148:123317. 29604840 10.1063/1.5009387

[50] Monachino E. , Jergic S., Lewis J.S., Xu Z.-Q., Lo A.T.Y., O'Shea V.L., Berger J.M., Dixon N.E., van Oijen A.M. A primase-induced conformational switch controls the stability of the bacterial replisome. Mol. Cell. 2020; 79:140–154. 32464091 10.1016/j.molcel.2020.04.037 PMC7335327

[51] Tanner N.A. , Loparo J.J., Hamdan S.M., Jergic S., Dixon N.E., van Oijen A.M. Real-time single-molecule observation of rolling-circle DNA replication. Nucleic Acids Res. 2009; 37:e27. 19155275 10.1093/nar/gkp006 PMC2651787

[52] Georgescu R.E. , Kurth I., O’Donnell M.E. Single-molecule studies reveal the function of a third polymerase in the replisome. Nat. Struct. Mol. Biol. 2011; 19:113–116. 22157955 10.1038/nsmb.2179 PMC3721970

[53] Georgescu R.E. , Yao N., Indiani C., Yurieva O., O’Donnell M.E. Replisome mechanics: lagging strand events that influence speed and processivity. Nucleic Acids Res. 2014; 42:6497–6510. 24829446 10.1093/nar/gku257 PMC4041431

[54] Graham J.E. , Marians K.J., Kowalczykowski S.C. Independent and stochastic action of DNA polymerases in the replisome. Cell. 2017; 169:1201–1213. 28622507 10.1016/j.cell.2017.05.041 PMC5548433

[55] Bailey S. , Eliason W.K., Steitz T.A. Structure of hexameric DnaB helicase and its complex with a domain of DnaG primase. Science. 2007; 318:459–463. 17947583 10.1126/science.1147353

[56] Carney S.M. , Gomathinayagam S., Leuba S.H., Trakselis M.A. Bacterial DnaB helicase interacts with the excluded strand to regulate unwinding. J.Biol. Chem. 2017; 292:19001–19012. 28939774 10.1074/jbc.M117.814178 PMC5704481

[57] Bujalowski W. , Jezewska M.J. Interactions of *Escherichia coli* primary replicative helicase DnaB protein with single-stranded DNA. The nucleic acid does not wrap around the protein hexamer. Biochemistry. 1995; 34:8513–8519. 7612593 10.1021/bi00027a001

[58] Arai K. , Kornberg A. A general priming system employing only *dnaB* protein and primase for DNA replication. Proc. Natl. Acad. Sci. U.S.A. 1979; 76:4308–4312. 228295 10.1073/pnas.76.9.4308 PMC411563

[59] Michel B. , Sandler S.J. Replication restart in bacteria. J. Bacteriol. 2017; 199:e00102-17. 28320884 10.1128/JB.00102-17 PMC5472809

[60] Windgassen T.A. , Wessel S.R., Bhattacharyya B., Keck J.L. Mechanisms of bacterial DNA replication restart. Nucleic Acids Res. 2018; 46:504–519. 29202195 10.1093/nar/gkx1203 PMC5778457

[61] Wu C.A. , Zechner E.L., Reems J.A., McHenry C.S., Marians K.J. Coordinated leading- and lagging-strand synthesis at the *Escherichia coli* DNA replication fork. V. Primase action regulates the cycle of Okazaki fragmant synthesis. J. Biol. Chem. 1992; 267:4074–4083. 1740453

[62] Yuan Q. , McHenry C.S. Cycling of the *E. coli* lagging strand polymerase is triggered exclusively by the availability of a new primer at the replication fork. Nucleic Acids Res. 2014; 42:1747–1756. 24234450 10.1093/nar/gkt1098 PMC3919610

[63] Fang L. , Davey M.J., O’Donnell M. Replisome assembly at *oriC*, the replication origin of *E. coli*, reveals an explanation for initiation sites outside an origin. Mol. Cell. 1999; 4:541–553. 10549286 10.1016/s1097-2765(00)80205-1

[64] Carr K.M. , Kaguni J.M. Stoichiometry of DnaA and DnaB Protein in initiation at the *Escherichia coli* chromosomal origin. J. Biol. Chem. 2001; 276:44919–44925. 11551962 10.1074/jbc.M107463200

[65] Marszalek J. , Kaguni J.M. DnaA protein directs the binding of DnaB protein in initiation of DNA replication in *Escherichia coli*. J. Biol. Chem. 1994; 269:4883–4890. 8106460

[66] Sutton M.D. , Carr K.M., Vicente M., Kaguni J.M. *Escherichia coli* DnaA protein. The N-terminal domain and loading of DnaB helicase at the *E. coli* chromosomal origin. J. Biol. Chem. 1998; 273:34255–34262. 9852089 10.1074/jbc.273.51.34255

[67] Seitz H. , Weigel C., Messer W. The interaction domains of the DnaA and DnaB replication proteins of *Escherichia coli*. Mol. Microbiol. 2000; 37:1270–1279. 10972842 10.1046/j.1365-2958.2000.02096.x

[68] Felczak M.M. , Simmons L.A., Kaguni J.M. An essential tryptophan of *Escherichia coli* DnaA protein functions in oligomerization at the *E. coli* replication origin. J. Biol. Chem. 2005; 280:24627–24633. 15878847 10.1074/jbc.M503684200

[69] Keyamura K. , Abe Y., Higashi M., Ueda T., Katayama T. DiaA dynamics are coupled with changes in initial origin complexes leading to helicase loading. J. Biol. Chem. 2009; 284:25038–25050. 19632993 10.1074/jbc.M109.002717 PMC2757208

[70] Kim S. , Dallmann H.G., McHenry C.S., Marians K.J. Coupling of a replicative polymerase and helicase: a τ–DnaB interaction mediates rapid replication fork movement. Cell. 1996; 84:643–650. 8598050 10.1016/s0092-8674(00)81039-9

[71] Yuan Q. , Dohrmann P.R., Sutton M.D., McHenry C.S. DNA polymerase III, but not polymerase IV, must be bound to τ-containing DnaX complex to enable exchange into replication forks. J. Biol. Chem. 2016; 291:11727–11735. 27056333 10.1074/jbc.M116.725358 PMC4882441

