# Supplementary material for: DnaB helicase dynamics in bacterial DNA replication resolved by single-molecule studies
Source: Nucleic Acids Res. 2021 Jun 17;49(12):6804–16. doi: 10.1093/nar/gkab493 (PMC8266626; doi:10.1093/nar/gkab493)
Supplement: gkab493_Supplemental_File [file gkab493_supplemental_file.pdf]

SUPPLEMENTARY DATA

DnaB helicase dynamics in bacterial DNA replication resolved by single-molecule studies

Richard R. Spinks[1,2], Lisanne M. Spenkelink[1,2], Sarah A. Stratmann[3], Zhi-Qiang Xu[1,2], N. Patrick J. Stamford[4], Susan E. Brown[4], Nicholas E. Dixon[1,2,4], Slobodan Jergic[1,2], and Antoine M. van Oijen[1,2]

[1]Molecular Horizons and School of Chemistry and Molecular Bioscience, University of Wollongong, Wollongong, New South Wales 2522, Australia,

[2]Illawarra Health & Medical Research Institute, Wollongong, New South Wales 2522, Australia,

[3]Zernike Institute for Advanced Materials, University of Groningen, Groningen 9747 AG, the Netherlands,

[4]Research School of Chemistry, Australian National University, Canberra, Australian Capital Territory 2601, Australia

SUPPLEMENTARY METHODS

Plasmids and strains

The *E. coli recA* strain AN1459 (S1), grown at 30˚C in LB media, was used as host during plasmid construction, and strain BL21(λDE3)*recA* (S2) was used for preparative over-production of the $DnaB_6(DnaC)_6$ complex. The *dnaC*⁺ plasmid pJK129 (S3) and the *dnaB*⁺ plasmid pKA1 (S4) were gifts of Dr Arthur Kornberg (Stanford University). The vector pPT150 (S5) contains tandem phage λ p_R and p_L promoters upstream of a synthetic ribosome-binding site (RBS) perfectly complementary to the 3' end of 16*S* rRNA and a unique *Hpa*I restriction site, as well as unique *Nco*I and *Sma*I sites located ~350 bp further downstream. Vector pPT150 also expresses the λ *c*I*857*^{ts} allele; the *c*I*857* protein represses the λ promoters at 30˚C but is inactivated at 42˚C, enabling temperature-induced overexpression of target genes.

Overproduction of DnaC directed by plasmids pPS237 and pSB957

A 2634-bp *Nco*I–*Hpa*I fragment containing the intact *dnaC* and *yjjA* genes was isolated from pJK129 and inserted between the *Nco*I and *Sma*I sites of pPT150. The new plasmid pPS231 (6297 bp) was linearised with *Nco*I and treated with sufficient exonuclease *Bal*31 to remove ~285 bp of DNA upstream of the ATG start codon of *dnaC* (and a similar amount from the other end). The product mixture was digested with *Hpa*I and plasmids recircularised by intramolecular ligation, a strategy designed to bring the synthetic RBS in pPT150 into close proximity to the ATG start codon of *dnaC*. Since moderate overproduction of DnaC protein is lethal to *E. coli*, transformants were first screened for a temperature-sensitive (*ts*) phenotype on replica plates at 42˚C, then plasmids in 96 *ts* strains were subjected to high-resolution restriction mapping to identify those likely to promote highest-level overproduction of DnaC.

Nucleotide sequence determination and small-scale temperature induction experiments with SDS-PAGE analysis led to retention of pPS237 (Supplementary Figure S1A), a plasmid that directed high-level overproduction of DnaC at 42˚C. Unfortunately, most of the DnaC protein was found not to be in the soluble fraction on cell lysis. To facilitate construction of plasmid pSB958 (see below), pPS237 was linearised with *Nde*I, the overhanging ends were filled in with Pol I (Klenow) and dNTPs, and the plasmid religated to yield pSB957 (Supplementary Figure S1A)

Overproduction of DnaB directed by plasmid pPS359

An ~4.4-kb *Bam*HI–*Hin*dIII *dnaB*$^{+}$*npt*$^{+}$(kan^{R}) fragment from plasmid pKA1 was inserted between the same sites in vector pUC9 (S6); transformants were selected at 30˚C for resistance to ampicillin and checked for kanamycin resistance and complementation of the *dnaB*^{ts} mutation in strain SG1692*recA* (S7). The ~2.8 kb *dnaB*$^{+}$ *Nde*I fragment from the product plasmid pPS307 was treated with sufficient *Bal*31 to remove ~44 bp from each end, then digested with *Eco*RI. The ~2.1 kb *dnaB*$^{+}$ fragments were isolated and inserted between the *Hpa*I and *Eco*R1 sites of pPT150. Ninety-six ampicillin-resistant transformants were screened for a *ts* phenotype at 42˚C, and eight selected plasmids were restriction mapped with *Bam*HI and *Nco*I. These transformants were then screened for overproduction of DnaB using small-scale cultures and SDS-PAGE, followed by nucleotide sequence determination to demonstrate the proximity of the strong RBS from pPT150 to the ATG start codon of *dnaB*. Finally, a small *Sma*I fragment was removed from the selected plasmid pPS353 to yield plasmid pPS359 (Supplementary Figure S1B). Although pPS359 directed high-level overproduction of DnaB, the protein also largely remained in the insoluble fraction after cell lysis.

Overproduction of the DnaBC complex directed by plasmids pPS562 and pSB958

Because of the poor solubility of DnaB and DnaC in their respective overproducing strains, we developed methods for their co-expression in near-stoichiometric amounts by constructing synthetic *dnaCB* operons under control of the tandem phage λ promoters in the parent vectors (Supplementary Figure S1C). Plasmid pPS237 (Supplementary Figure S1A) contains a unique *Acc*I site just following the TAA stop codon of *dnaC*; it was linearised with *Acc*I and treated with Pol I (Klenow) and dNTPs to fill in the ends, before being further digested with *EcoRI* and the large (4769-bp) fragment isolated. Concurrently, plasmid pPS359 (Supplementary Figure S1B) was digested with *Bam*HI and the ends filled with Pol I (Klenow) before being further digested with *Eco*RI. The 2122-bp *dnaB*$^{+}$ fragment was isolated and ligated to the *dnaC*$^{+}$ vector fragment from pPS237 (see above), to yield pPS562 (Supplementary Figure S1C). Finally, to simplify production of mutant forms of *dnaB* and *dnaC* (S2,S8,S9), the unique *Acc*I site downstream of *dnaC* in pPS562 was restored, and an *Nde*I site was generated at the ATG start codon of *dnaB* to generate plasmid pSB958 (Supplementary Figure S1C). This was accomplished by ligation of the 4771-bp *Acc*I–*Eco*RI fragment of pSB957 (Supplementary Figure 1A), the 2006-bp *Pvu*I–*Eco*R1 fragment of pPS359 (Supplementary Figure S1B) and a 111-bp *Acc*I–*Pvu*I fragment generated by PCR using primers 310 (5’-CCAGCAGATCTTCGCTGGTACG

CCCCTGCG-3'; within *dnaB*) and 311 (5'-TTCTGGCGGTAGTATACTAAGGAGGTTTCCATATGGC AGGAAATAAACCCTTC-3'; *Acc*I and *Nde*I sites underlined), followed by endonuclease digestion with *Acc*I and *Pvu*I. Both plasmids pPS562 and pSB958 have been used interchangeably for overproduction of wild-type DnaB and DnaC proteins, in soluble form.

Purification of DnaB and DnaC

Buffers used were: lysis buffer (50 mM Tris.HCl pH 7.6, 10% w/v sucrose, 200 mM NaCl, 3 mM EDTA, 2 mM dithiothreitol, 20 mM spermidine); buffer A (30 mM Tris.HCl pH 7.6, 10 mM $MgCl_2$, 2 mM dithiothreitol, 100 μM ADP, 20% v/v glycerol); buffer B (25 mM Tris.HCl pH 7.6, 1 mM dithiothreitol, 10% v/v glycerol); buffer C (50 mM Tris.HCl pH 7.6, 200 mM NaCl, 5 mM $MgCl_2$, 2 mM dithiothreitol, 100 μM ATP, 20% v/v glycerol); buffer D (40 mM Tris.HCl pH 7.6, 100 mM NaCl, 10 mM $MgCl_2$, 2 mM dithiothreitol, 100 μM ATP, 20% v/v glycerol); buffer E (50 mM Tris.HCl pH 7.6, 5 mM $MgCl_2$, 2 mM dithiothreitol, 1 mM ATP, 20% v/v glycerol).

E. coli strain BL21(λDE3)recA/pSB958 was grown at 30˚C in LB medium supplemented with thymine (50 μg ml^{-1}) and ampicillin (200 μg ml^{-1}). Upon growth to A_{595} = 0.8–1.0, the temperature was rapidly increased to 42˚C to induce overproduction of DnaB and DnaC; the 1-litre cultures were shaken for a further 3 h, after which they were chilled in ice. Cells were harvested by centrifugation (11,000 × *g*, 5 min), frozen in liquid nitrogen and stored at –80˚C.

Frozen cells (12.3 g from 4 litres of culture) were resuspended in 370 ml of ice-cold lysis buffer by gentle agitation. Then a solution of 100 mg of egg lysozyme in 10 ml of water was added dropwise, while stirring. After the suspension had been stirred gently at 4˚C for 60 min, it was warmed in a bath at 37˚C for 6 min (with gentle inversion each minute). Complete C protease inhibitor pills (Roche) were then added to the suspension as per manufacturer's prescription and then phenylmethylsulfonyl fluoride, dissolved in ethanol to 100 mM, was added dropwise to the agitated suspension to reach a final concentration of 0.5 mM. The suspension was rapidly cooled in ice, and then gently stirred for another 60 min at 4˚C. Cell debris was removed by centrifugation (35,000 × *g*, 30 min). Solid ammonium sulphate (0.21 g per ml) was added to the supernatant (Fraction I) over the course of 30 min, and left to stir for an additional 30 min. The pellet was harvested by centrifugation (35,000 × *g*, 30 min), dissolved in 30 ml of buffer A + 25 mM NaCl, and dialysed against three changes of 2 litres of the same buffer, to yield Fraction II.

The dialysate was loaded at 1 ml min^{-1} onto a column (2.5 × 16 cm) of Toyopearl DEAE-650M anion-exchange resin that had been equilibrated in buffer A + 25 mM NaCl. The column was washed with 100 ml of the same buffer, after which a linear gradient (500 ml) of 0–500 mM NaCl in buffer A was applied at a flow rate of 1.5 ml min^{-1}. Fractions that did not bind to the column containing DnaC (flow-through, 90 ml) were pooled and dialysed against buffer B (Fraction III). On the other hand, DnaB eluted in a single peak at ~230 mM NaCl. Fractions containing DnaB (50 ml, ~110 mg of protein) were pooled judiciously to avoid contamination by nucleic acids. DnaB in these fractions was precipitated by the

addition of solid ammonium sulphate (0.4 g ml^{-1}) and dissolved in 12 ml of buffer C (Fraction IV).

Fraction III (containing DnaC) was loaded at 1 ml min^{-1} onto a column (2.5 × 10 cm) of Toyopearl SP-650M cation-exchange resin that had been equilibrated in buffer B. The column was washed with 60 ml of buffer B at a flow rate of 1 ml min^{-1}, after which DnaC was eluted in a linear gradient (500 ml) of 0–500 mM NaCl in buffer B in a peak at ~200 mM NaCl. Fractions containing DnaC (34 ml) were pooled, dialysed into storage buffer D, frozen in liquid nitrogen and stored at –80˚C. A typical preparation yielded ~35 mg of highly purified DnaC.

To separate DnaB from contaminating DNA, Fraction IV was divided into two equal portions (6 ml each) that were separately loaded at 0.5 ml min^{-1} onto a column (2.5 × 65 cm) of Sephacryl S-400 gel filtration resin (GE Healthcare), which has been equilibrated in buffer C. DnaB eluted in the included volume at ~220 ml, whereas most of DNA (and aggregated species) eluted in the excluded volume at ~130 ml. Fractions containing DnaB (36 ml from each run) were pooled, frozen in liquid nitrogen and stored at –80˚C. A typical preparation yielded ~80 mg of highly purified DnaB.

Assembly and purification of the DnaB$_6$(DnaC)$_6$ (or DnaBC) complex

To assemble the DnaBC complex, 18.6 ml of DnaB (at 1.18 mg ml^{-1}) and 13.4 ml of DnaC (at 1.48 mg ml^{-1}) were gently mixed into 44 ml of buffer E that had been supplemented with an additional 1.5 ml of ATP stock solution (25 mM). These conditions provided an ~1.7-fold molar excess of DnaC over DnaB (calculated each as monomers) in a solution finally containing 1 mM ATP and <70 mM NaCl. The solution was stirred gently at 4˚C for 60 min, then clarified by centrifugation (35,000 × *g*; 30 min) and finally loaded at 0.5 ml min^{-1} onto a onto a column (1 × 6 cm) of Toyopearl Super Q-650M anion-exchange resin that had been equilibrated in buffer E + 80 mM NaCl. After the column had been washed with 30 ml of buffer E + 80 mM NaCl at a flow rate of 1.5 ml min^{-1}, the DnaBC complex was eluted in a steep linear gradient (45 ml) of 80–1400 mM NaCl in buffer E in a peak centred at ~180 mM NaCl (Supplementary Figure S1D). Fractions containing DnaBC were pooled (7.5 ml in total), frozen in liquid nitrogen and stored at –80˚C. This preparation yielded ~24 mg of highly purified DnaBC complex.

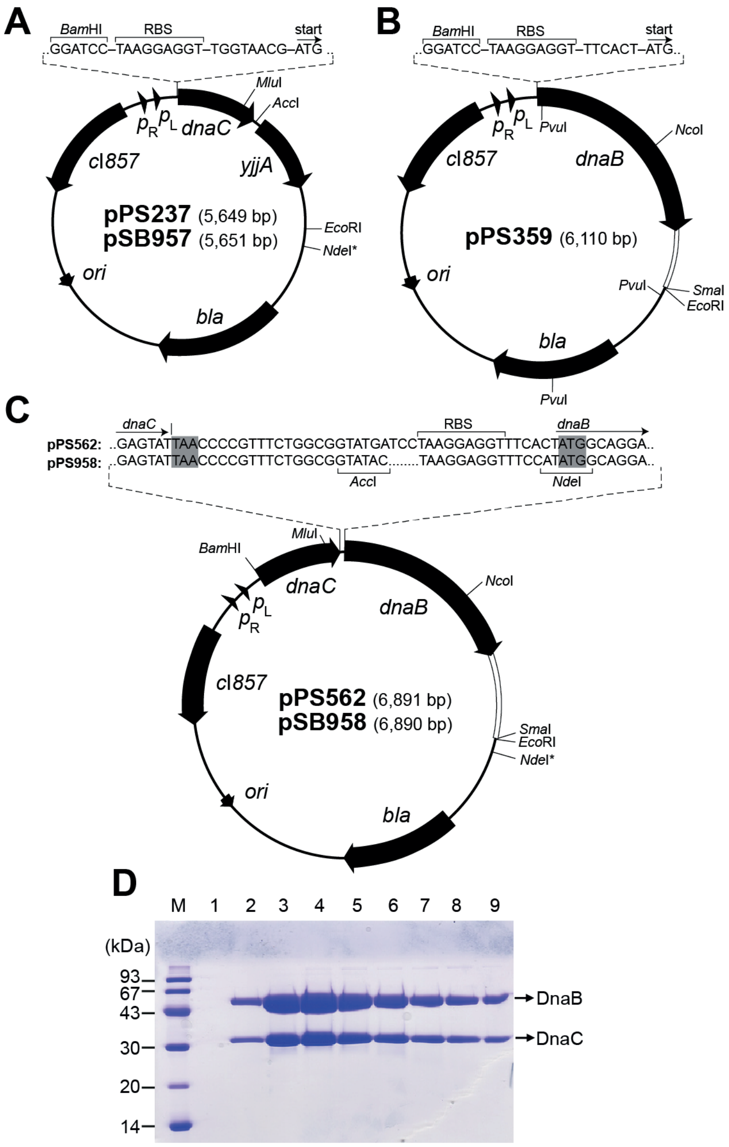

Supplementary Figure S1. Genetic and physical maps of plasmids that direct overproduction of DnaB and DnaC and isolation of the DnaBC complex. (**A**) The *dnaC*⁺ plasmids pPS237 and pSB957. The restriction site marked *Nde*I* is present in pPS237, but absent from pSB957. (**B**) The *dnaB*⁺ plasmid pPS359. (**C**) Plasmids pPS562 and pSB958, containing synthetic *dnaCB* operons that direct simultaneous overproduction of both DnaB and DnaC, in near stoichiometric amounts. The restriction site marked *Nde*I* is present in pPS562, but absent from pSB958. (**D**) Purification of the DnaB.DnaC complex on a 5-ml Super Q-650M anion-exchange column. Samples from successive fractions (indicated by numbers) were analysed on a 15% SDS-PAGE gel.

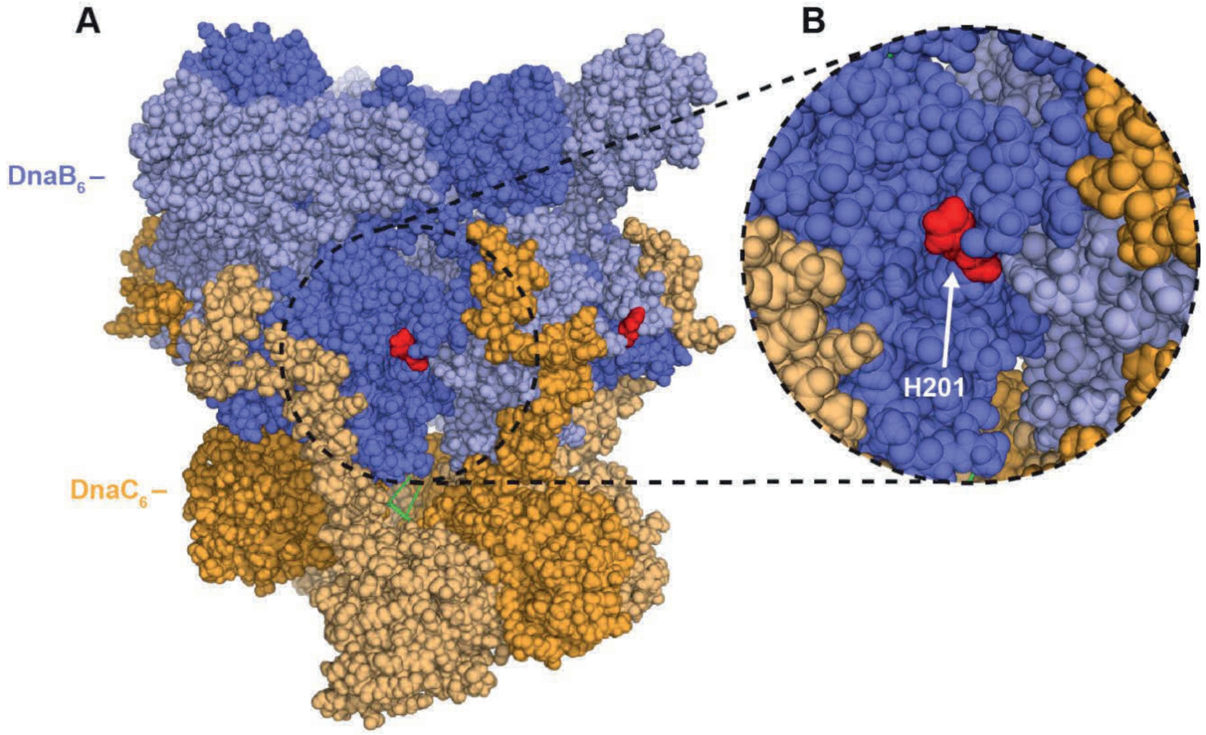

Supplementary Figure S2. Structural model (PDB ID: 6QEM) of the *E. coli* DnaB helicase (blue shades) in complex with the DnaC helicase loader (orange shades) highlighting the position of the H201C mutation used for mutagenesis and labelling (red) (S10). (**A**) Side view of the $DnaB_6(DnaC)_6$ hexameric 'cracked ring' conformation bound to ssDNA (green; mostly buried). (**B**) Close up view of the His201 residue positioned on the linker between the C- and N-terminal domains of DnaB protomers. This residue is not involved with DnaC or DnaG primase binding (not shown), and is solvent exposed in a surface loop, making it suitable for mutation to cysteine for fluorescent labelling.

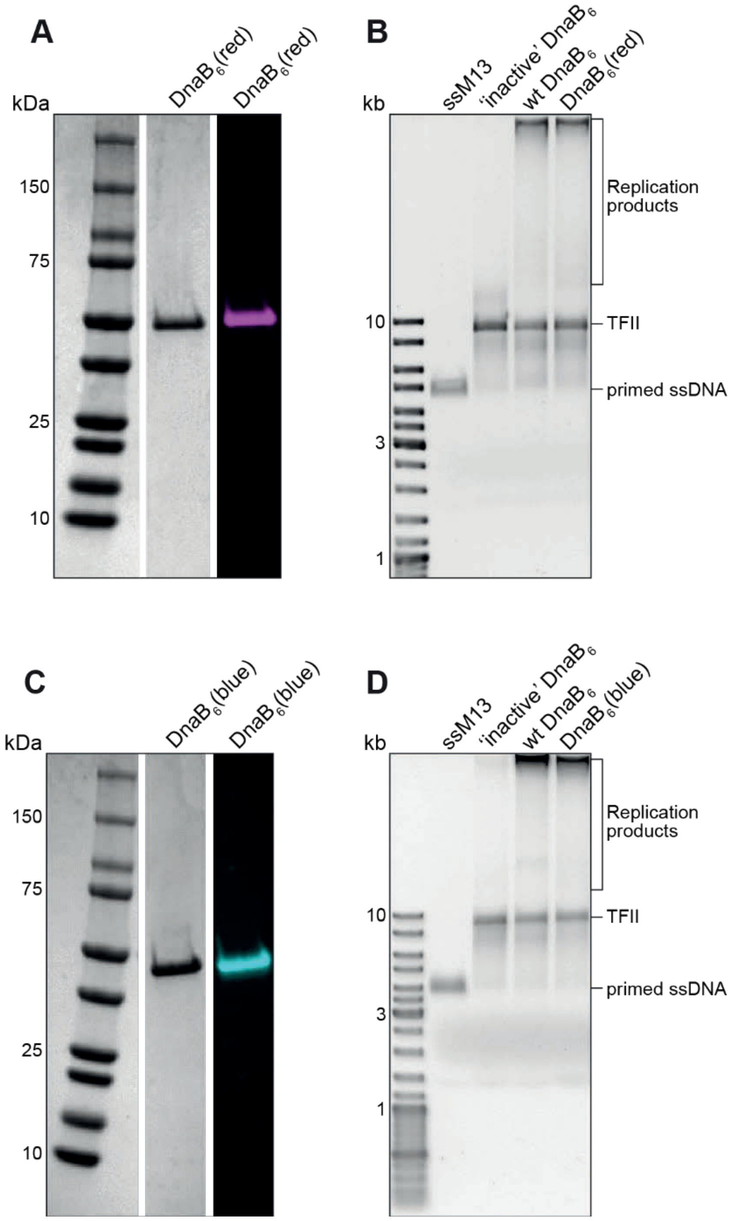

Supplementary Figure S3. Activity of the labelled DnaB helicases. (**A**) SDS-PAGE gel of pure DnaB$_6$(red). The left and middle lane are stained with Coomassie blue and imaged using a Bio-Rad Gel Doc XR, while the right lane is unstained and the Alexa Fluor 647 fluorescence is imaged using an Amersham Imager 600. (**B**) Comparison of the activities of inactive DnaB$_6$ (from a preparation that resulted in inactive DnaB$_6$, apparently due to transition-metal ion contamination), WT DnaB$_6$ and DnaB$_6$(red). Native agarose gel electrophoresis separates the products of leading-strand DNA replication from filled-in primed M13 (TFII) and unreplicated primed ssM13. The replication products are dependent on active DnaB and there is no difference between the WT DnaB$_6$ and DnaB$_6$(red). (**C**) SDS-PAGE of pure DnaB$_6$(blue) similar to (A). (**D**) Native agarose gel electrophoresis similar to (B) showing the activity of DnaB$_6$(blue).

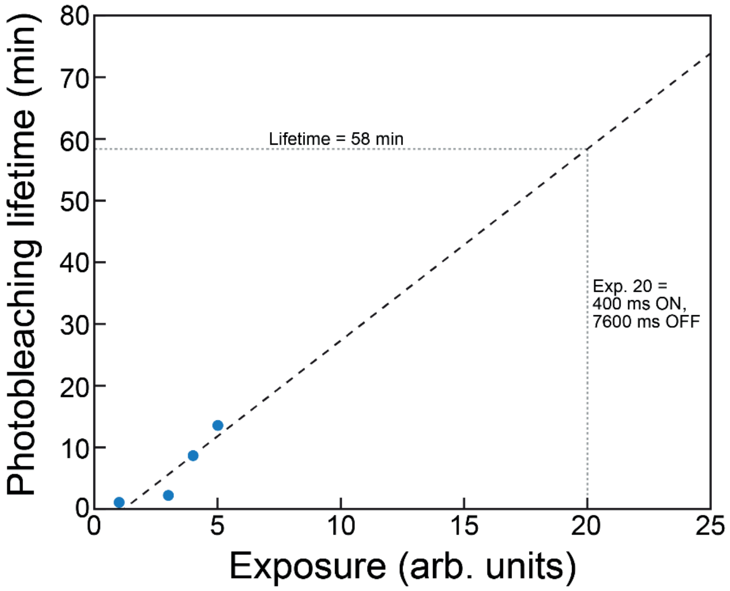

Supplementary Figure S4. Photobleaching lifetime extrapolation. The photobleaching lifetime of $DnaB_6$(red) was measured at four separate extents of laser exposure, where the laser power was constant at 40 W/cm^{-2} and the increasing *x*-axis represents longer intermission between exposure periods. Fitting these points with a linear function and extrapolating to exposure ‘20’ (400 ms ON, 7600 ms OFF) provides a measure of the photobleaching lifetime of ~58 min under these conditions.

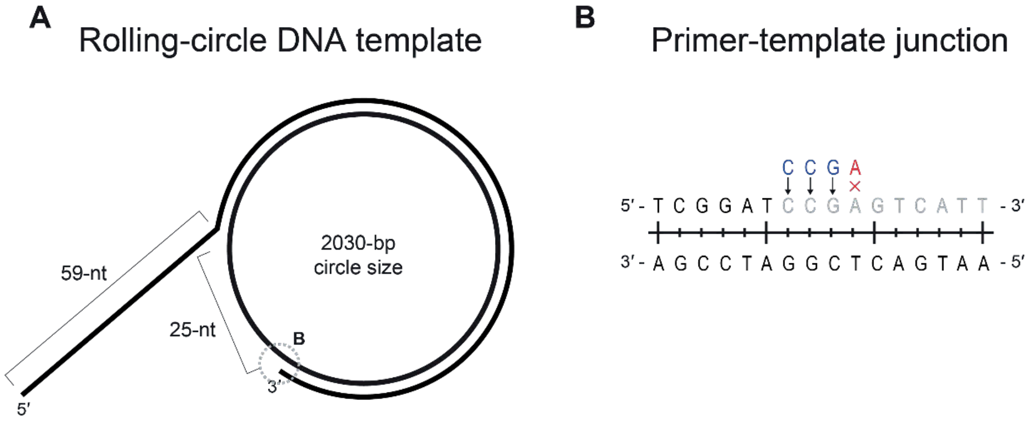

Supplementary Figure S5. The rolling-circle DNA template with controlled fork topology. (**A**) The rolling-circle DNA template was created as previously described (S11) by modifying a 2030-bp plasmid with an oligonucleotide with non-complementary region creating a 59-nt 5′-tail. This tail forms a replication fork suitable for loading of the DnaBC complex. The modification also leaves a 25-nt gap where the leading-strand Pol III core can associate with the primer–template junction. (**B**) The primer–template junction in (A). The base-pair composition of this junction is important in the stationary replisome association assay, wherein dATP and dTTP are omitted from the association solution, which allows the Pol III core to bind the junction, incorporate the first three nucleotides (CCG) but not the fourth (A). Thus, the polymerase is retained at the junction cycling between the polymerisation and proofreading states.

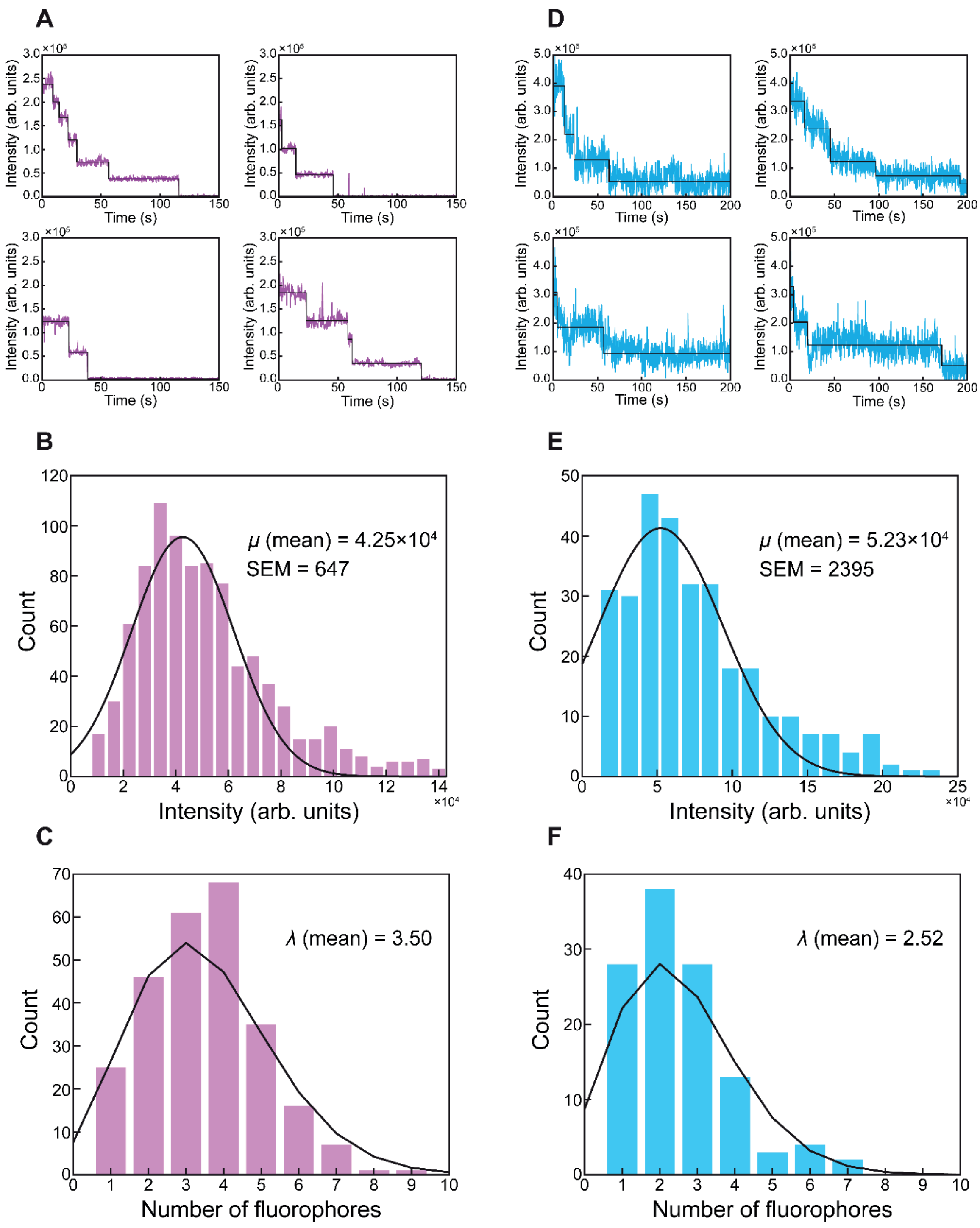

Supplementary Figure S6. Quantification of the degree of labelling through single-molecule photobleaching steps. Both DnaB$_6$(red) and DnaB$_6$(blue) contain multiple fluorescent dyes per hexamer; however, the presence of ADP in the storage buffer interferes with measurement of the degree of labelling by spectrophotometry. Therefore, we deposited (**A**) DnaB$_6$(red) or (**D**) DnaB$_6$(blue) from 20 pM solutions of each on a cleaned glass coverslip and single-molecule photobleaching steps (black lines) were detected using change-point analysis (S12–S14). The distribution of step size for (**B**) DnaB$_6$(red) was (4.25 ± 0.06) x 10^4 (μ (mean) ± S.E.M., n = 909) and for (**E**) DnaB$_6$(blue) was (5.23 ± 0.24) x 10^4 (μ (mean) ± S.E.M., n = 293). (**C**) Distribution of the number of steps and thus number of dyes per hexamer, for DnaB$_6$(red). The black line is a Poisson fit of the data (λ (mean) = 3.50, n = 260). (**F**) Distribution of the number of steps and thus number of dyes per hexamer for DnaB$_6$(blue). The black line is a Poisson fit of the data (λ (mean) = 2.52, n = 181).

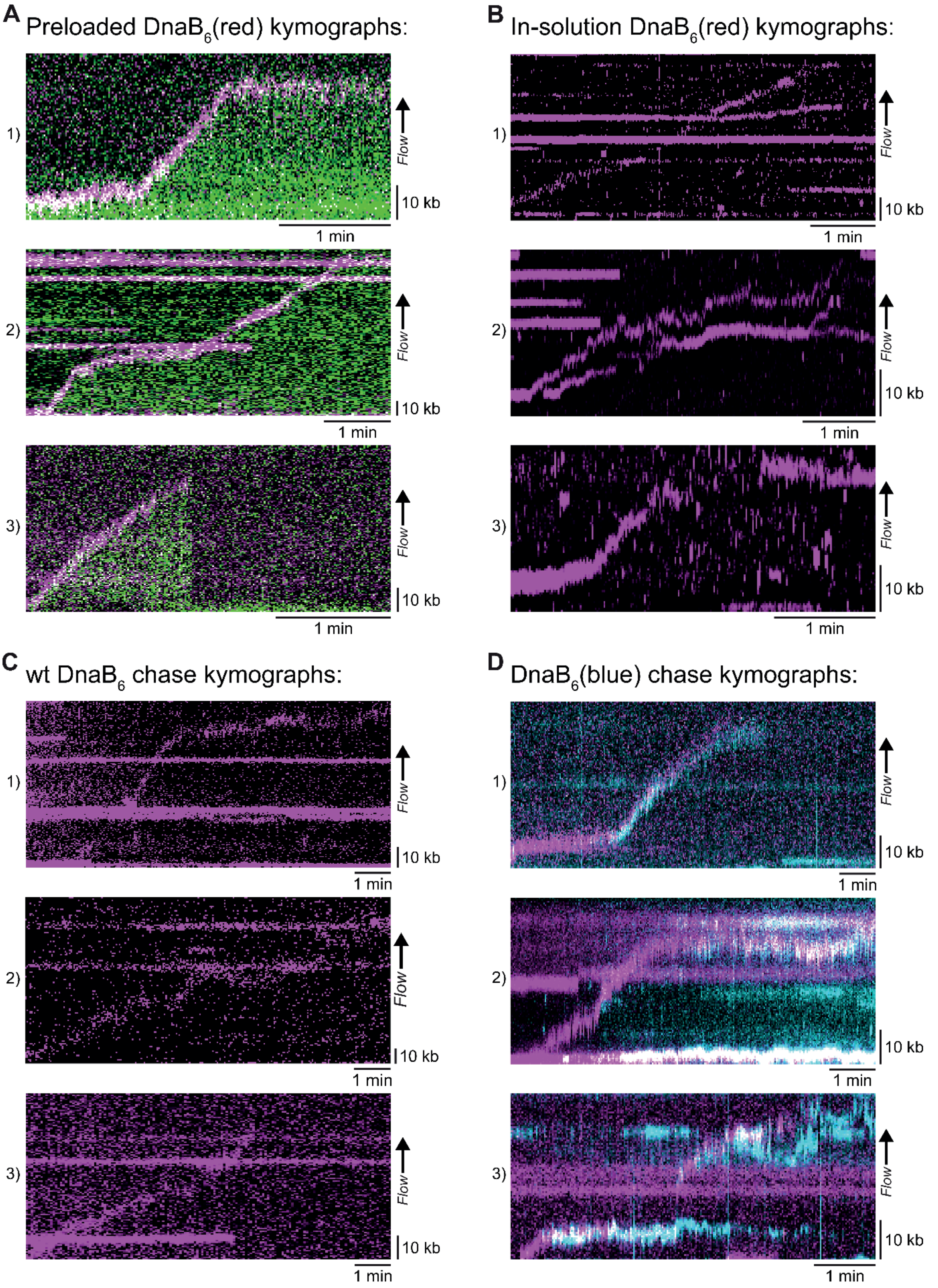

Supplementary Figure S7. Further examples of kymographs from the rolling-circle replication assays. (**A**) Kymographs of preloaded $DnaB_6$(red) overlaid with SYTOX Orange-stained DNA (green) during rolling-circle replication. (**B**) Kymographs of $DnaB_6$(red) moving at the fork during rolling-circle replication when excess $DnaB_6$(red) is present in solution. (**C**) Kymographs of $DnaB_6$(red) moving with the fork during rolling-circle replication whilst chased with WT $DnaB_6$. (**D**) Kymographs of $DnaB_6$(red) as it is chased with $DnaB_6$(blue) as they are moving with the fork during rolling-circle replication.

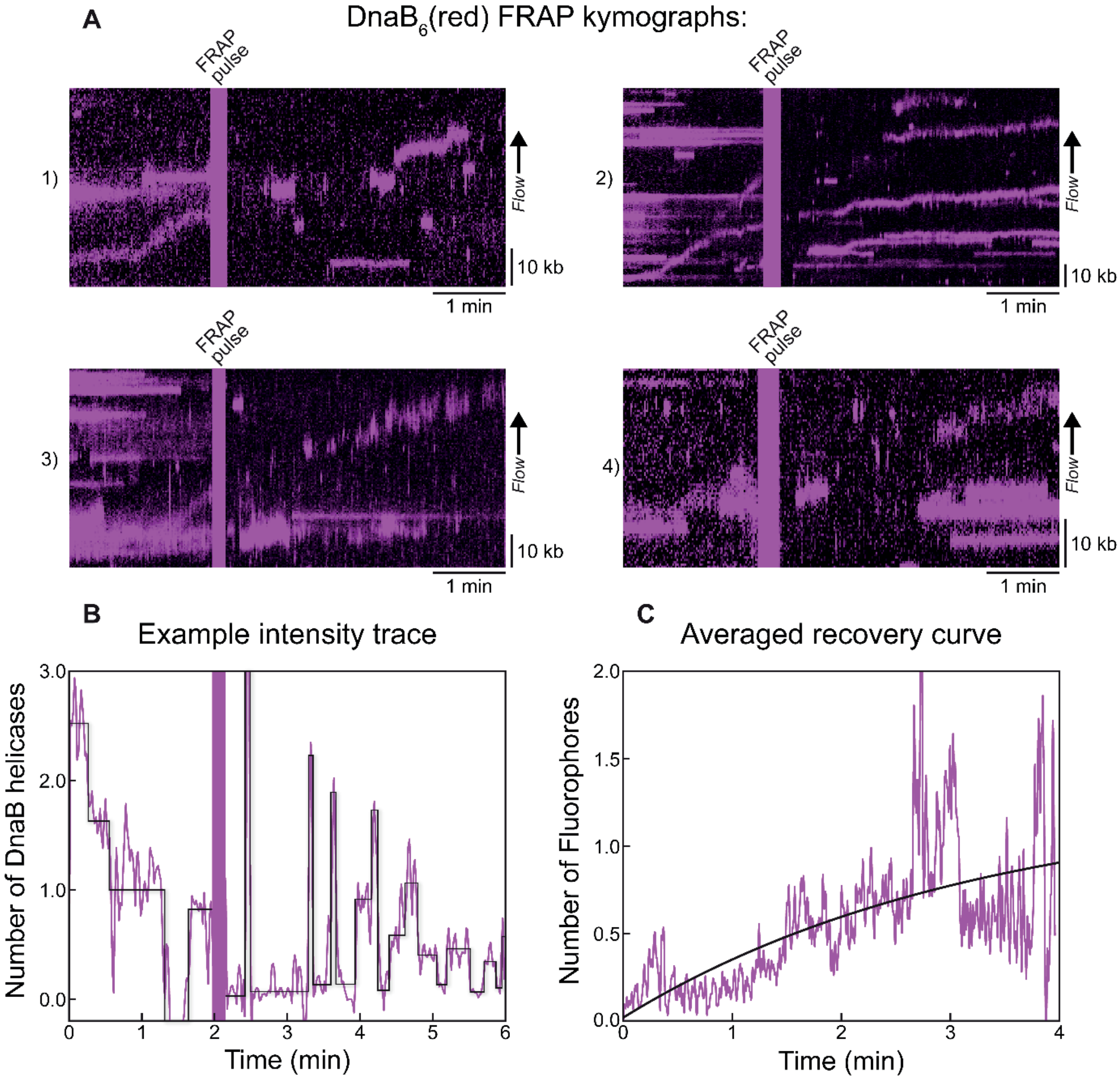

Supplementary Figure S8. DnaB helicase dynamics quantified by single-molecule FRAP. (**A**) Example kymographs of $DnaB_6$(red) (2 nM) in the single-molecule FRAP experiments during rolling-circle replication. After 2 min of replication, a high intensity FRAP pulse (240 W cm^{-2}; indicated by the magenta line) is used to rapidly bleach all $DnaB_6$(red) foci in the field of view. Recovery dynamics are observed over the following 4 min. (**B**) Number of DnaB helicases as a function of time corresponding to the above kymograph A3, where single-molecule steps (black line) were detected using change-point analysis (S12–S14). (**C**) The average intensity recovered by 21 molecules of replicating $DnaB_6$(red) in the single-molecule FRAP experiments. Fitting these data with a FRAP recovery function (Equation 2) determines a steady state of 0.34 ± 0.06 helicases at the fork (or 1.2 ± 0.2 fluorophores; mean ± S.E.M) and a characteristic exchange time of 2.8 ± 0.7 min (mean ± S.E.M.). Despite obtaining these values, the poor fit of this model (*SSE* = 81.5, R^2 = 0.4478) suggests the kinetics measured here represent transient association rather than exchange.

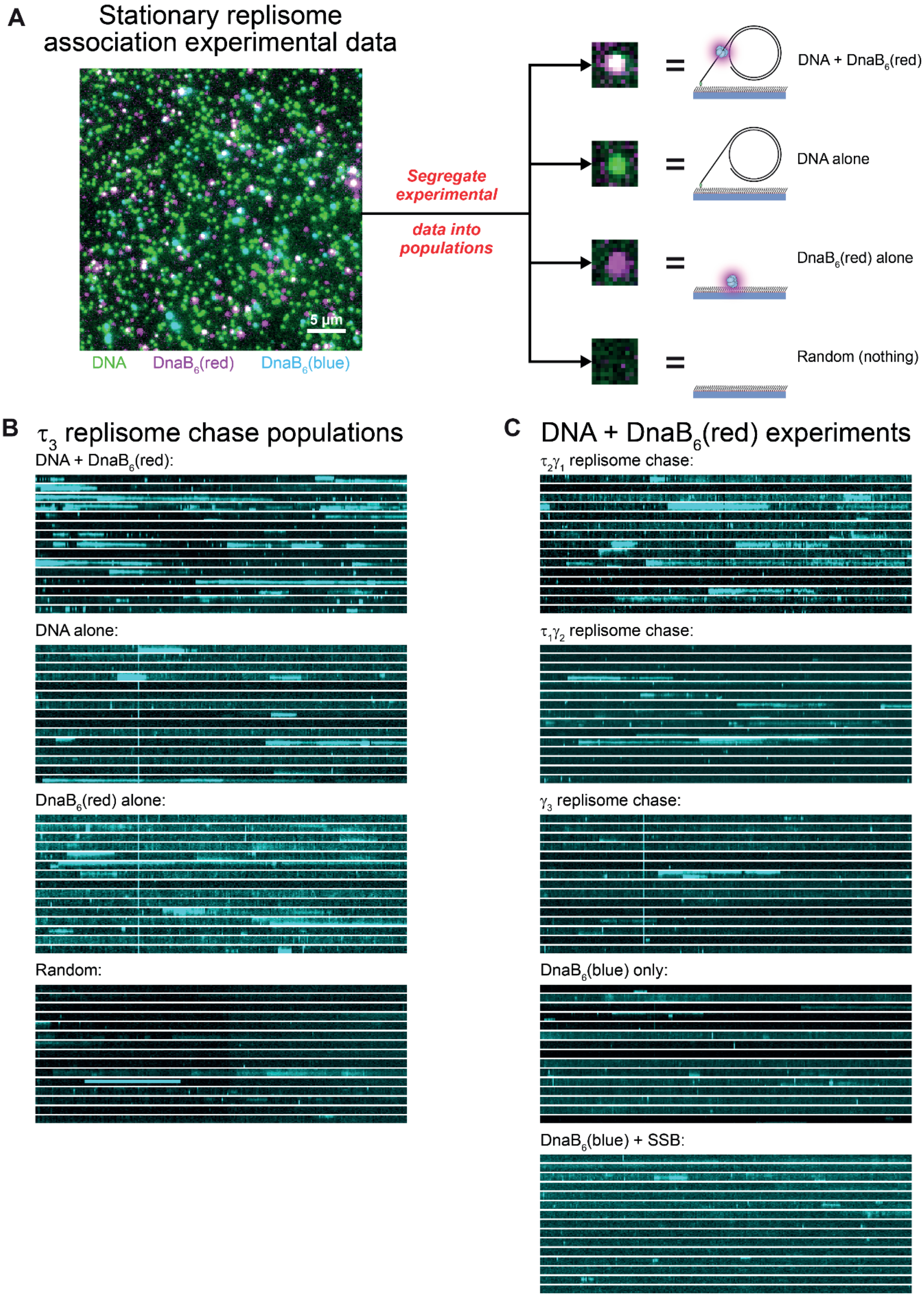

Supplementary Figure S9. Analysis of the stationary replisome association experiments. (**A**) The analysis pipeline to process the stationary replisome association experiments (left) and to identify different populations within the data (right). (**B**) Fifteen example $DnaB_6$(blue) kymographs for each population extracted from the τ_3 replisome chase experiment. (**C**) Fifteen example $DnaB_6$(blue) kymographs for the DNA-$DnaB_6$(red) population from different experiments.

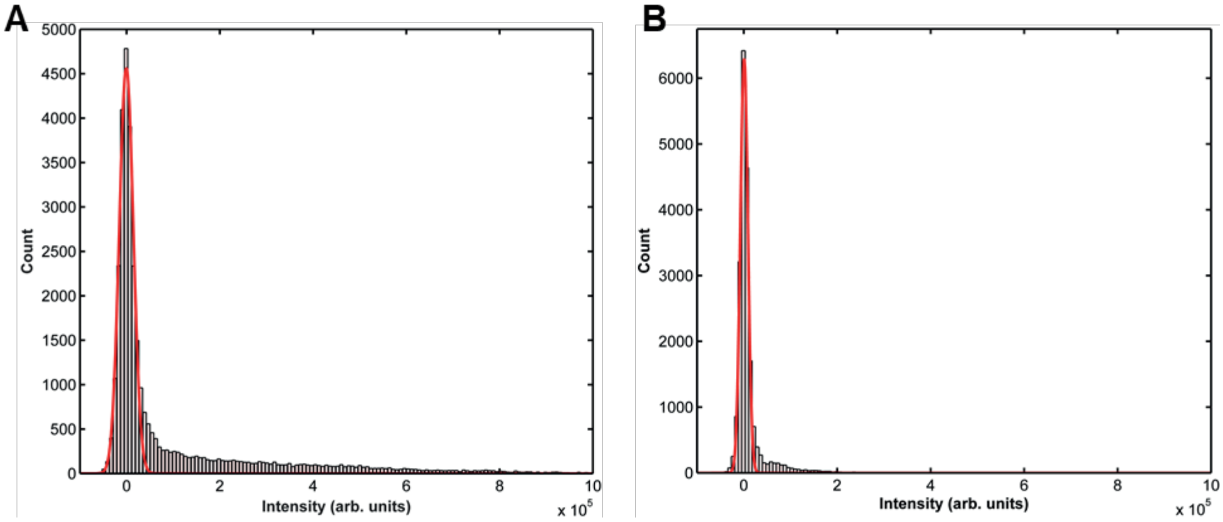

Supplementary Figure S10. Steady state analysis of the stationary replisome association experiments. (**A**) Histogram of all the intensity points recorded for all the $DnaB_6$(blue) trajectories in the DNA + $DnaB_6$(red) condition from the τ_3 replisome chase experiment (n = 123). All the points excluded to the right of the zero-point Gaussian fit (red line) represent the fraction of time spent with $DnaB_6$(blue) bound (43%). (**B**) Histogram of all the intensity points recorded for all the $DnaB_6$(blue) trajectories in the DNA + $DnaB_6$(red) condition from the $DnaB_6$(blue) only chase experiment (n = 43). In this case, the fraction of time spent with $DnaB_6$(blue) bound is less (12%).

A

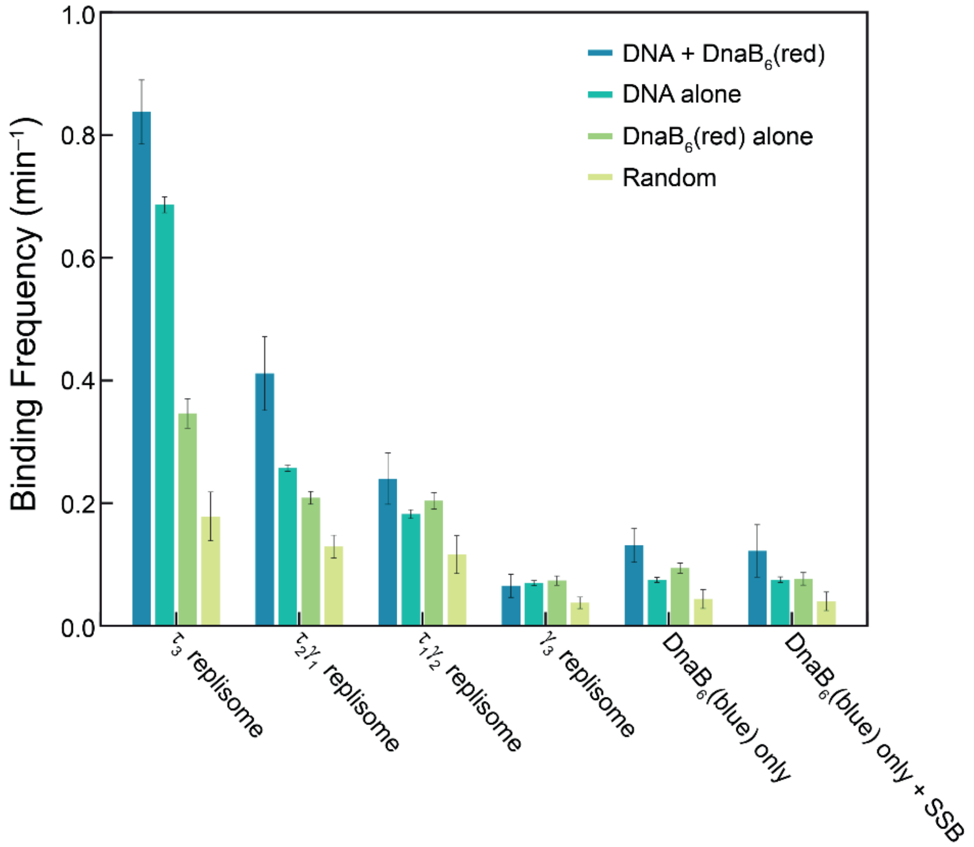

B

	DNA + $DnaB_6$(red)	DNA alone	$DnaB_6$(red) alone	Random
τ_3 **replisome**	0.84 ± 0.05 (123)	0.69 ± 0.01 (1651)	0.35 ± 0.02 (320)	0.18 ± 0.04 (37)
$\tau_2\gamma_1$ **replisome**	0.41 ± 0.06 (26)	0.26 ± 0.01 (1256)	0.21 ± 0.01 (239)	0.13 ± 0.02 (45)
$\tau_1\gamma_2$ **replisome**	0.24 ± 0.04 (70)	0.18 ± 0.01 (1489)	0.20 ± 0.01 (504)	0.12 ± 0.03 (37)
γ_3 **replisome**	0.07 ± 0.02 (43)	0.07 ± 0.01 (1328)	0.07 ± 0.01 (351)	0.04 ± 0.01 (32)
**$DnaB_6$(blue) only**	0.13 ± 0.03 (74)	0.07 ± 0.01 (1510)	0.09 ± 0.01 (421)	0.04 ± 0.02 (32)
**$DnaB_6$(blue) + SSB**	0.12 ± 0.04 (36)	0.08 ± 0.01 (1385)	0.08 ± 0.01 (252)	0.04 ± 0.02 (40)

mean ± S.E.M (*n*)

Supplementary Figure S11. Comparison of stationary replisome association data. (**A**) Graphical representation of all the measured binding frequencies for different experiments (plotted along the *x*-axis) and different populations within these experiments (colour corresponding to the legend). (**B**) Tabulation of binding frequencies (units in min^{-1}) for the different conditions corresponding to the experiments in (A). The number of molecules is indicated in brackets.

SUPPLEMENTARY REFERENCES

S1. Vasudevan,S.G., Armarego,W.L.F., Shaw,D.C., Lilley,P.E., Dixon,N.E. and Poole,R.K. (1991) Isolation and nucleotide sequence of the *hmp* gene that encodes a haemoglobin-like protein in *Escherichia coli* K-12. *Mol. Gen. Genet.*, **226**, 49–58.

S2. Williams,N.K., Prosselkov,P., Liepinsh,E., Line,I., Sharipo,A., Littler,D.R., Curmi,P.M.G., Otting,G. and Dixon,N.E. (2002) *In vivo* protein cyclization promoted by a circularly-permuted *Synechocystis* sp. PCC6803 DnaB mini-intein. *J. Biol. Chem.*, **277,** 7790–7798

S3. Kobori,J.A. and Kornberg,A. (1982) The *Escherichia coli dnaC* gene product. I. Overproduction of the dnaC proteins of *Escherichia coli* and *Salmonella typhimurium* by cloning into a high copy number plasmid. *J. Biol. Chem.*, **257**, 13757–13762.

S4. Nakayama,N., Arai,N., Bond,M.W., Kaziro,Y. and Arai, K.-I. (1984) Nucleotide sequence of *dnaB* and the primary structure of the *dnaB* protein from *Escherichia coli. J. Biol. Chem.*, **259**, 97–101.

S5. Elvin,C.M., Thompson,P.R., Argall,M.E., Hendry,P., Stamford,N.P.J., Lilley,P.E. and Dixon,N.E. (1990) Modified bacteriophage lambda promoter vectors for overproduction of proteins in *Escherichia coli*. *Gene*, **87**, 123–126.

S6. Vieira,J. and Messing,J. (1982) The pUC plasmids, an M13mp7-derived system for insertion mutagenesis and sequencing with synthetic universal primers. *Gene*, **19,** 259–268.

S7. Lilley,P.E., Stamford,N.P.J., Vasudevan,S.G. and Dixon,N.E. (1993) The 92-min region of the *Escherichia coli* chromosome: Location and cloning of the *ubiA* and *alr* genes. *Gene*, **129**, 9–16.

S8. Williams,N.K., Liepinsh,E., Watt,S.J., Prosselkov,P., Matthews,J.M., Attard,P., Beck,J.L., Dixon,N.E. and Otting, G. (2005) Stabilization of native protein fold by intein-mediated covalent cyclization. *J. Mol. Biol.*, **346**, 1095–1108.

S9. Watt,S.J., Urathamakul,T., Schaeffer,P.M., Williams,N.K., Sheil,M.M., Dixon,N.E. and Beck, J.L. (2007) Multiple oligomeric forms of *Escherichia coli* DnaB helicase revealed by electrospray ionization mass spectrometry. *Rapid Commun. Mass Spectrom.*, **21,** 132–140.

S10. Arias-Palomo,E., Puri,N., O'Shea Murray,V.L., Yan,Q. and Berger,J.M. (2019) Physical basis for the loading of a bacterial replicative helicase onto DNA. *Mol. Cell*, **74**,173–184.e4.

S11. Monachino,E., Ghodke,H., Spinks,R.R., Hoatson,B.S., Jergic,S., Xu,Z.-Q., Dixon,N.E. and van Oijen,A.M. (2018) Design of DNA rolling-circle templates with controlled fork topology to study mechanisms of DNA replication. *Anal. Biochem.*, **557**, 42–45.

S12. Watkins,L.P. and Yang,H. (2005) Detection of intensity change points in time-resolved single-molecule measurements. *J. Phys. Chem. B*, **109**, 617–628.

S13. Duderstadt,K.E., Geertsema,H.J., Stratmann,S.A., Punter,C.M., Kulczyk,A.W., Richardson,C.C. and van Oijen,A.M. (2016) Simultaneous real-time imaging of leading and lagging strand synthesis reveals the coordination dynamics of single replisomes. *Mol. Cell*, **64**, 1035–1047.

S14. Hill,F.R., van Oijen,A.M. and Duderstadt,K.E. (2018) Detection of kinetic change points in piece-wise linear single molecule motion. *J. Chem. Phys.*, **148**, 123317.
